# Transcriptional Basis of Ca^2+^ Remodeling Reversal Induced by Polyamine Synthesis Inhibition in Colorectal Cancer Cells

**DOI:** 10.3390/cancers15051600

**Published:** 2023-03-04

**Authors:** Enrique Pérez-Riesgo, Elena Hernando-Pérez, Verónica Feijóo, Sendoa Tajada, Lucía Núñez, Carlos Villalobos

**Affiliations:** 1 Unidad de Excelencia Instituto de Biomedicina y Genética Molecular de Valladolid (IBGM), Universidad de Valladolid and Consejo Superior de Investigaciones Científicas (CSIC), 47003 Valladolid, Spain; 2 Departamento de Bioquímica y Biología Molecular y Fisiología, Facultad de Medicina, Universidad de Valladolid, 47005 Valladolid, Spain

**Keywords:** colorectal cancer, polyamines, intracellular calcium, transcriptomic analysis, DFMO, store-operated calcium entry, mitochondria, TRP channels

## Abstract

**Simple Summary:**

The common activation of the c-Myc oncogene in colorectal cancer (CRC) induces the overexpression of ornithine decarboxylase (ODC), the limiting step in polyamine synthesis, which is a process blocked by α-Difluoromethylornithine (DFMO), an ODC suicide inhibitor and potential CRC treatment. We showed previously that intracellular Ca^2+^ homeostasis is remodeled in CRC and contributes to cancer hallmarks. We investigated whether polyamine synthesis inhibition induced by DFMO may reverse this remodeling in CRC and, if so, the molecular basis for this phenotypic reversal. To this end we used calcium imaging and transcriptomic analysis in both normal and CRC cells. We found that CRC cells showed enhanced resting Ca^2+^ and store-operated Ca^2+^ entry (SOCE) but decreased Ca^2+^ store content relative to normal cells. Polyamine synthesis inhibition reversed not only this Ca^2+^ remodeling but also reversed the changes in the transcription of a dozen genes involved in Ca^2+^ transport in CRC cells, including genes modulating Ca^2+^ entry into the cells as store-operated channels and TRP channels as well as Ca^2+^ extrusion systems in the plasma membrane and mitochondria. These results provide a molecular basis for the role of polyamine synthesis in Ca^2+^ remodeling in cancer.

**Abstract:**

Colorectal cancer (CRC) is associated with mutations in APC/Wnt leading to c-myc activation and the overexpression of ODC1, the limiting step in polyamine synthesis. CRC cells also display a remodeling of intracellular Ca^2+^ homeostasis that contributes to cancer hallmarks. As polyamines may modulate Ca^2+^ homeostasis during epithelial tissue repair, we investigated whether polyamine synthesis inhibition may reverse Ca^2+^ remodeling in CRC cells and, if so, the molecular basis for this reversal. To this end, we used calcium imaging and transcriptomic analysis in normal and CRC cells treated with DFMO, an ODC1 suicide inhibitor. We found that polyamine synthesis inhibition partially reversed changes in Ca^2+^ homeostasis associated with CRC, including a decrease in resting Ca^2+^ and SOCE along with an increased Ca^2+^ store content. We also found that polyamine synthesis inhibition reversed transcriptomic changes in CRC cells without affecting normal cells. Specifically, DFMO treatment enhanced the transcription of SOCE modulators CRACR2A; ORMDL3; and SEPTINS 6, 7, 8, 9, and 11, whereas it decreased SPCA2, involved in store-independent Orai1 activation. Therefore, DFMO treatment probably decreased store-independent Ca^2+^ entry and enhanced SOCE control. Conversely, DFMO treatment decreased the transcription of the TRP channels TRPC1 and 5, TRPV6, and TRPP1 while increasing TRPP2, thus probably decreasing Ca^2+^ entry through TRP channels. Finally, DFMO treatment enhanced the transcription of the PMCA4 Ca^2+^ pump and mitochondrial channels MCU and VDAC3 for enhanced Ca^2+^ extrusion through the plasma membrane and mitochondria. Collectively, these findings suggested the critical role of polyamines in Ca^2+^ remodeling in colorectal cancer.

## 1. Introduction

Colorectal cancer (CRC) is one of the most common types of cancer and causes of cancer deaths worldwide, with nearly 1,250,000 new CRC cases every year and a mortality rate as high as 50% of all cases [1]. The molecular basis of CRC involves in most cases the activation of the Wnt–β-catenin signaling pathway by the mutation of intracellular components such as APC, AXIN, and CTNNB1/β-catenin genes or the epigenetic alteration of Wnt inhibitors such as DKK-1, SFRPs, and WIF, considered the initial steps in colorectal tumorigenesis [2]. These changes result in the activation of c-myc and K-ras, which lead to adenoma, adenocarcinoma, and colon carcinoma [2]. Myc activation induces the overexpression of multiple genes, including ornithine decarboyxlase (ODC), the limiting step in the synthesis of the polyamines putrescine, spermine, and spermidine. ODC activation can be efficiently prevented by the suicide inhibitor Difluoromethylornithine (DFMO, also named eflornithine) [3]. Substantial evidence links ODC overexpression, excess polyamines synthesis, and CRC. For instance, ODC is overexpressed in most CRCs, and different tumor promoters induce ODC1 and tumor formation [4,5]. ODC polymorphisms have also been reported in CRC. In addition, targeting ODC and polyamines using cell lines, animal models, and even clinical trials may efficiently prevent CRC [6,7,8]. For instance, DFMO inhibits colon carcinogenesis in ApcMin/+ mice with increased levels of ODC and polyamines in intestinal tissues and suppresses carcinogenesis in the small intestines of these mice [3,4]. Interestingly, the grade of intestinal polyps is polyamine-dependent, and the anti-carcinogenic effects can be rescued by putrescine. DFMO may work in humans as well. An ongoing trial is presently evaluating the effectiveness of the combination of DFMO and sulindac in preventing colon adenomas [9]. However, the mechanisms by which polyamines promote carcinogenesis remain to be fully established.

Polyamines are physiological molecules that are produced transiently during epithelial restitution for epithelial tissue repair. This process involves the transient activation of cell migration and/or proliferation after wounding. Evidence suggests that this process could be mediated by Ca^2+^ signaling induced by changes in the expression of TRPC1 channels as well as an increased ratio of STIM1 to STIM2 [10]; molecular players involved in SOCE in epithelial cells, mediated by caveolin [11]; and the small guanosine-5′-triphosphate-binding protein RhoA [12]. Interestingly, SOCE and its molecular players have recently been found to be involved in carcinogenesis in CRC and other forms of cancer [13,14,15]. For instance, we recently reported that intracellular Ca^2+^ homeostasis is remodeled in CRC cells [14,16,17]. Specifically, CRC cells display enhanced SOCE and decreased Ca^2+^ store content relative to normal colonic cells, and these changes contribute to cancer hallmarks, such as increased cell proliferation, cell invasion, and resistance to apoptosis [16,17]. At the molecular level, enhanced SOCE was linked to the increased expression of TRPC1 and an increased ratio of STIM1 to STIM2 in CRC cells [16,17], thus mimicking changes previously reported to be induced by transient polyamine exposure during epithelial restitution [10]. In addition, we also reported that store-operated currents (SOCs) are quite different in normal and colon cancer cells. Specifically, normal colonic cells display typical CRAC-like currents driven by Orai1 channels, which are very small, Ca^2+^-selective, inward-rectifying currents. In contrast, CRC cells display larger, non-selective currents with both inward and outward components that are mediated by both Orai1 and TRPC1 channels [16]. We recently conducted a transcriptomic analysis of 77 selected gene transcripts involved in intracellular Ca^2+^ transport that provided the first insights into the transcriptional basis of this remodeling [18]. In short, we found the differential expression of selected voltage-operated Ca^2+^ channels and SOCE players, transient receptor potential (TRP) channels, Ca^2+^ release channels, Ca^2+^ pumps, Na^+^/Ca^2+^ exchanger isoforms, and genes involved in mitochondrial Ca^2+^ transport [18]. Therefore, the evidence suggests that intracellular Ca^2+^ homeostasis is largely remodeled in CRC, and these changes could be mediated by excess polyamine synthesis linked to CRC.

To address this issue, we recently tested the effects of polyamine synthesis inhibition on Ca^2+^ remodeling in CRC cells [19]. In accordance with this hypothesis, we reported that CRC cells overexpressed ODC1 relative to normal cells. In addition, polyamine synthesis inhibition in CRC cells that were resistant to cell death reversed this phenotype and sensitized CRC cells to apoptosis. Importantly, polyamine synthesis inhibition promoted changes in intracellular Ca^2+^ homeostasis consistent with phenotype reversal, including changes in store-operated currents and SOCE, Ca^2+^ store content, and the expression of a few proteins involved in SOCE [19]. However, whether polyamine synthesis inhibition reverses the whole calcium signature linked to carcinogenesis remains to be addressed.

Here, we combined calcium imaging and transcriptomic analysis using next-generation sequencing and microarray technology to determine the molecular basis of Ca^2+^ remodeling in CRC and the effects of polyamine synthesis inhibition on transcriptomic remodeling and changes in intracellular Ca^2+^ homeostasis. Our results indicated that polyamine synthesis inhibition partially reversed the remodeling of intracellular Ca^2+^ in CRC cells. In addition, polyamine synthesis inhibition induced expression changes in 25% of the whole transcriptome of CRC cells but had nearly negligible effects on normal cells. Finally, we found that the reversal of Ca^2+^ remodeling depended on the changes in a dozen genes, including SOCE modulators, several TRP channels, two Ca^2+^ pumps, and two channels involved in mitochondrial Ca^2+^ transport.

## 2. Materials and Methods

### 2.1. Materials

The HT29 cell line was obtained from LONZA (Basel, Switzerland). NCM460 cells were from INCELL Corporation (San Antonio, TX, USA). SW480 cells were a kind gift from Prof. Alberto Muñoz (CSIC, Madrid, Spain). Dulbecco’s modified Eagle’s medium (DMEM), penicillin, streptomycin, and fetal bovine serum (FBS) were sourced from Lonza (Basel, Switzerland). L-glutamine was from Gibco (Barcelona, Spain). Trypsin-EDTA was from LONZA (Verbiers, Belgium). Poly-L-Lysin was from Marlenfeld GmbH (Lauda-Könlgshofen, Germany). Six-well plates were from NUNC (Thermo Scientific, Waltham, MA, USA). Dishes 10 cm^2^ in diameter were from Corning (NY, USA). DFMO was from TOCRIS (Bristol, UK). Fura2/AM and qPCR primers are from Invitrogen (Eugene, OR, USA). Cyclopiazonic acid (CPA) was from Sigma-Aldrich (Steinheim, Alemania). Antibodies against MCU and β actin were from Sigma (Madrid, Spain). The RNA extraction kit was a GeneMATRIX Universal RNA Purification Kit from EURx (Gdansk, Poland). Clariom D human microarrays (Affymetrix) were supplied by CABIMER (Andalucía, Spain). RNA-seq (Illumina) was provided by Sistemas Genómicos S.L (Valencia, Spain). PolyamineRED was from Funakoshi Co., Ltd., Tokyo, Japan). All other reagents were obtained from Sigma and Merck.

### 2.2. Cell Models and Sample Preparation

As human colon cancer models, we used the HT29 and SW480 colon cancer cell lines, and the NCM460 cell line was employed as the normal control; all of these have been widely used and validated in the colon cancer research field. Cells were cultured in a 25 cm^2^ flask with DMEM plus 1 g/L of glucose, 10% FBS, 1% penicillin/streptomycin, and 1% L-glutamine, which were placed into an incubator at 37 °C under a 10% CO_2_ atmosphere. All cells were used in passage 2. From each cell culture seeded in one flask, cells were detached with trypsin-EDTA and broken into two parts. One of these parts, which was used for the calcium imaging experiment, was seeded on four glass coverslips pretreated with poly-L-Lysin; each of them was then placed into a well in a 6-well plate with a cell density of 3000 cells per coverslip. The other part, which was used for the transcriptomic experiment (microarrays), was seeded on two dishes 10 cm^2^ in diameter with a cell density of 10.5 × 10^5^ cells per dish. Notably, two flasks, one flask of HT29 and the other of NCM460 cells, were processed on the same day. Then, two flasks (one from HT29 cell cultures and the other from NCM460) were processed every day on four different days.

**Figure 1:**
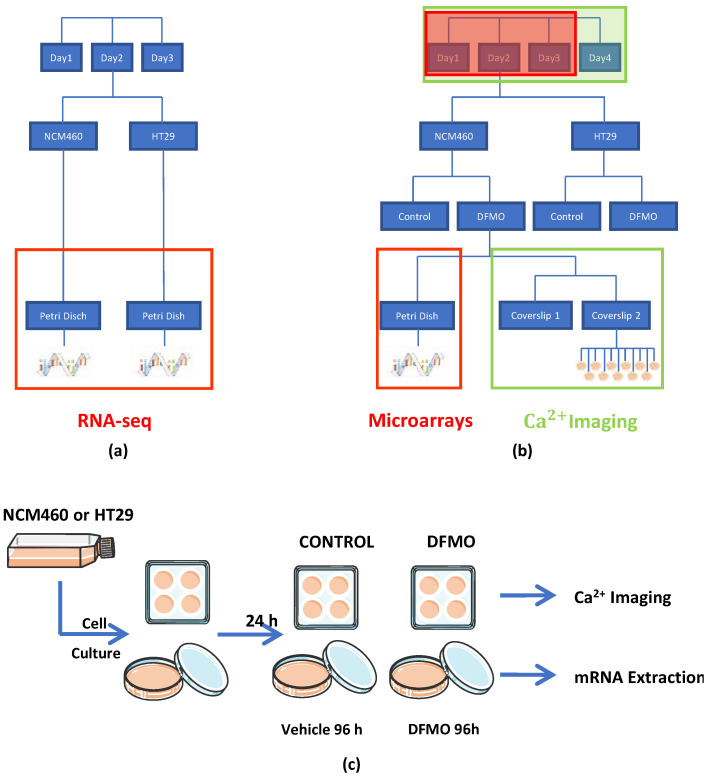
Transcriptomics and functional experimental designs. (**a**) For each cellular line, the mRNA was extracted, isolated, and analyzed with Illumina technology. Specifically, in a single day (up to a total of 3 days), one sample from every cellular line was processed. (**b**,**c**) For every cellular line, we seeded 4 coverslips into 6-well plates and 2 dishes of 10 cm. After 24 h, treatment was added (control or DFMO 5 mM). After 96 h, on the one hand, the mRNA from the samples seeded into dishes was extracted and isolated to carry out transcriptomic assays. On the other hand, samples seeded over coverslips into 6-well plates were used for Ca^2+^ imaging experiments.

After an entire day at 37 °C under a 10% CO_2_ atmosphere, of the eight coverslips and four dishes obtained from the two flasks (with one flask per cell line), two coverslips and one dish from each flask were treated with DFMO (DFMO 5 mM in DMEM 1 g/L glucose plus 10% dialyzed FBS), whereas the other two coverslips and dish from each flask were used to represent the non-treated conditions, i.e., treated without DFMO (DMEM 1 g/L glucose plus 10% dialyzed FBS). Then, the eight coverslips and four dishes were kept in an incubator at 37 °C under a 10% CO_2_ atmosphere for 96 h. Next, the coverslips were used for calcium imaging experiments, and the dishes were used to extract and isolate their mRNA for transcriptomic analysis (see procedure schematic in Figure 1a–c). Notice that a transcriptome analysis was previously carried out for both the NCM460 and HT29 cell lines without DFMO treatment using Illumina RNA-seq for comparison.

### 2.3. Intracellular Polyamine Levels

**Figure 2:**
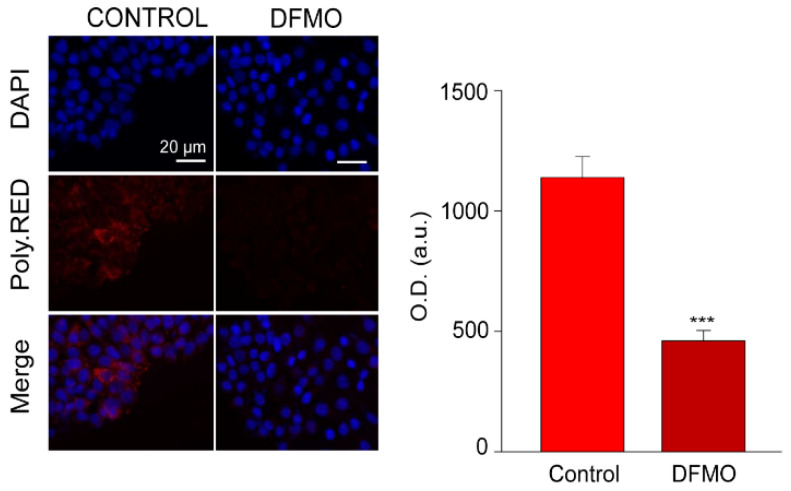
Effect of DFMO treatment on intracellular polyamine levels in HT29 cells. Intracellular polyamine levels were estimated by measuring the red fluorescence of PolyamineRED (PolyRED) reagent on non-treated (control) or DFMO-treated cells. Images are representative of experiments on HT29 cells in the same acquisition conditions. Bar plot displays values corresponding to optical density (O.D.) from each cell, and the background was removed. The sample size was equal to 4 coverslips for each set of experimental conditions. The *p*-value corresponding to the hypothesized contrast in polyamines levels between the control and DFMO-treated cells was lower than 0.001, so we rejected the null hypothesis and accepted that polyamines levels were reduced in cells treated with DFMO. *** *p* < 0.001.

Intracellular polyamines were estimated using fluorescence imaging and PolyamineRED (Funakoshi Co., Ltd., Japan), an intracellular polyamine detection reagent. Treated and control cells were cultured with 30 µM of the reagent in free-serum media for 30 min according to manufacturer’s procedure. Then, cells were fixed, and nuclei were stained with DAPI. Fluorescence images were obtained using a Nikon Eclipse 90i fluorescence microscope and analyzed with ImageJ software. Figure 2 shows that PolyamineRED fluorescence in cells treated with DFMO was largely reduced relative to untreated cells, in accordance with polyamine depletion. Similar results were obtained in SW480 and NCM460 cells.

### 2.4. Experimental Design

The effect of the inhibition of the polyamine synthesis pathway through DFMO on both HT29 and NCM460 cells was assessed at both the transcriptomic and functional levels (Figure 1). On the one hand, the transcriptomic experiment was conducted using Clarion D human microarrays from Affymetrix and, on the other hand, the functional test was carried out using calcium imaging. Then, four experimental conditions were evaluated: NCM460 cells, both treated and non-treated with DFMO, and HT29 cells, again both treated and non-treated with DFMO. Furthermore, since we were interested in obtaining the transcriptomic expression profile through calcium imaging assays, we acquired from the same flask, at the same time, a replicate for every experimental condition (for the same cell line) and for both transcriptomic and calcium imaging assays, as shown in Figure 1c. Specifically, on the same day, a replicate for both treated and non-treated experimental conditions was extracted from the same flask, and two replicates for calcium imaging for the experimental condition “g” were obtained and processed from one replicate for the same experimental conditions in the transcriptomic experiment. Furthermore, prior to the experiments described above, both non-treated HT29 and NCM460 cells were assessed at the transcriptomic level through Illumina RNA-seq technology. Regarding the factors that needed to be considered for later data analysis, on the one hand, in the calcium imaging experiment, there were two random factors (day and coverslip) and another two fixed factors (cell line and treatment). Then, we had to use linear mixed models to control the effects of random factors. On the other hand, concerning the transcriptomic experiment, there were two fixed factors (cell line and treatment) and just one random factor. We took into account this random factor, since it was necessary to control it. The data analysis for each type of experiment is explained more in detail in the corresponding paragraph.

### 2.5. Single-Cell Calcium Measurements

Fluorescence calcium measurements were conducted in colon cancer HT29 and SW480 cells as well as normal NCM460 cells, all treated and non-treated with DFMO. First of all, cells were washed with a standard external medium (SEM) containing (in mM) NaCl 145, KCl 5, CaCl_2_ 1, MgCl_2_ 1, glucose 10, and Hepes/NaOH 10 (pH 7.4). Then, cells were loaded with 4 μM of Fura2/AM, the acetoxymethyl ester form of the calcium-sensitive dye fura2, in SEM for 45 min at room temperature and in the dark. After loading, coverslips with attached cells were mounted in the perfusion chamber of a Zeiss Axiovert 100 TV inverted microscope and perfused with prewarmed (37 °C) SEM. Then, the cells in the chamber were excited alternately by light at 340 and 380 nm using a xenon lamp and a filter wheel, and the light emitted by the cells at 520 nm was filtered through a dichroic mirror and recorded every 5 s by a Hamamatsu ER camera (Hamamatsu Photonics France). Finally, the 340 nm/380 nm ratios between each pair of pixels were calculated, and all those belonging to the same region of interest (ROI), corresponding to a single cell, were averaged and interpreted as an intracellular [Ca^2+^] measurement from that ROI, as detailed before [20,21]. For resting [Ca^2+^] measurements, we used the median 340 nm/380 nm ratio of the first 30 s from each cell. After that, to obtain a measure of the Ca^2+^ store content from each cell, cells were treated with the reversible sarcoplasmic and endoplasmic reticulum Ca^2+^ ATPase (SERCA) inhibitor cyclopiazonic acid (CPA) at 10 μM in a calcium-free SEM. Accordingly, the Ca^2+^ store content from each cell was measured as both the increment in and area under the curve (AUC) of the 340 nm/380 nm ratio for the signal corresponding to the rise in intracellular [Ca^2+^] induced by CPA in Ca^2+^-free medium. Finally, after store depletion, cells were perfused with SEM containing CPA and 1 mM Ca^2+^ to induce SOCE. Then, the SOCE corresponding to every single cell was measured as both the rise in and the area under the curve (AUC) of the F340 nm/F380 nm ratio for the signal corresponding to the rise in intracellular [Ca^2+^].

### 2.6. Transcriptomic Experiments

After RNA isolation (using a GeneMATRIX Universal RNA Purification Kit from EURx), microarrays were carried out in Centro Andaluz de Medicina Regenerativa (CABIMER, Seville, Spain), and RNA-seq assays were conducted by Sistemas Genómicos S.L (Valencia, Spain). Then, data analysis based on the microarrays was performed by our group using .CEL files, whereas data analysis for RNA-seq was performed using count-matrix, provided by Sistemas Genómicos S.L. (Valencia, Spain).

### 2.7. Western Blotting

HT29 cells were treated with vehicle or DFMO (5 mM, 96 h) and used for western blotting. Total protein was extracted from cells and used to quantify the expression of MCU. Whole-cell lysate was obtained using RIPA buffer (20 mM Tris–HCl, pH 7.8, 150 mM NaCl, 1% Triton X-100, 1% deoxycholic acid, 1 mM EDTA, 0.05% SDS) supplemented with the Halt™ Protease and Phosphatase Inhibitor Cocktail (100×) from ThermoFisher Scientific (ref #1861281) (Waltham, MC, USA). Protein concentrations were determined by a Bradford protein assay. Proteins were fractionated by SDS-PAGE; electroblotted onto PVDF membranes; and probed with the antibodies at a dilution of 1/200, except for the anti-β-actin, which was used at a dilution of 1/5000. The antibody against MCU (SC-246071) had been previously characterized and was visualized by the addition of goat anti-rabbit IgG or rabbit anti-mouse IgG. Detection was performed using Pierce ECL Western Blotting substrate (Thermo Scientific) and a VersaDoc Imaging System (BioRad, Munich, Germany). The quantification of protein expression was carried out using Quantity One software (BioRad, Munich, Germany). The datasets were analyzed by adjusting a linear model to fit the data, the model assumptions were evaluated, the response variables were transformed with Box–Cox family transformations because the normality assumption was violated, and outlier detection was carried out by an analysis of the Cook’s distance and the studentized residual. After transformation, the model assumptions were fulfilled as follows: residual normality with the Shapiro–Wilks test (*p* value = 0.1961) and homoscedasticity with the Bartlett test (*p* value = 0.8771). The sample size was equal to 6:3, corresponding to HT29 cells without treatment and HT29 cells treated with DFMO, respectively; *p* < 0.01.

### 2.8. RNA Isolation, RT, and Real-Time PCR

Total RNA from HT29 and NCM460 cells was isolated with a GeneMatrix Universal RNA purification kit (Eurx^®^, Molecular biology products) following the manufacturer’s instructions. A confluent 60 mm Petri dish per condition was employed for each assay. The quality of the RNA was determined by optical density measurements at 260 and 280 nm and by electrophoresis on agarose gels. After DNAse I treatment with a RapidOut DNA removal kit (Thermo Scientific), 1000 ng of RNA was reverse-transcribed using a High-Capacity cDNA Reverse Transcription Kit (Thermo Fisher Scientific) at 37 °C for 120 min to obtain cDNA.

Quantitative PCR was carried out on equal amounts of cDNA in triplicate for each sample using a Kapa Sybr^®^ Fast qPCR kit (Kapa Biosystems, Wilmington, MA, USA) in a LightCyler^®^ 480 (Roche, Basel, Switzerland) thermocycler. The primers used are shown in Table 1 and were designed using OligoPerfect Primer Designer (ThermoFisher Scientific). The cycling conditions were 5 min at 95 °C, 40 cycles of 95 °C for 15 s, and 60 °C for 20 s. These amplifications were used to compare the different samples and melting curves to determine the specificity of the PCR products.

The qPCR data were analyzed using the threshold cycle (Ct) relative quantification method (ΔΔCt). The gene expression levels were normalized by the housekeeping gene RP18S. The relative abundance of the genes was calculated as 2ΔCt, where ΔCt = Ctgene–Ct18S. Differences between cancer (HT29) and control (NCM460) cells were calculated from 2ΔΔCt, where ΔΔCt = ΔCt(HT29)–ΔCt(NCM460), using ΔCt(NCM460) as the calibrator. In this analysis, a value of 0 indicated no change, negative values indicated decreased expression, and positive values indicated increased expression relative to the calibrator. The statistical analysis method used was a Student’s t test with Bonferroni’s correction for two independent samples (ΔCt(HT29) and ΔCt(NCM460)) obtained with RP18S as endogenous control. * *p* < 0.05, ** *p* < 0.01, *** *p* < 0.001

### 2.9. Data Analysis: Calcium Imaging

For the calcium imaging experiments, we were interested in testing which experimental conditions were different from each other in terms of resting intracellular [Ca^2+^], Ca^2+^ store content, and SOCE. Thus, as response variables, at the single-cell level, we considered the median from the absolute value of the signal across the first 30 s for resting intracellular [Ca^2+^], and the corresponding increase in [Ca^2+^] levels and the area under the curve (AUC) for both Ca^2+^ store content and SOCE. We evaluated the effect of two factors, treatment and cellular line, on the response variable, each of them with just two levels (control and DFMO for the treatment factor, and NCM460 and HT29 for the cellular line factor), so there was a total of 4 experimental conditions: control-NCM460, DFMO-NCM460, control-HT29, and DFMO-HT29. Due to the experimental design, the dataset showed a hierarchical structure, since observations were clustered into, or organized at, different levels: the experiment was carried out on 4 different days; each day we performed 2 replicates corresponding to each of the 4 experimental conditions; and within each replicate, we measured several cells from the same experimental conditions. Furthermore, both replicates and day were random factors.

Since there were random factors and there could be a correlation between observations belonging to the same cluster (i.e., they were non-independent), it became necessary to use linear mixed models, also known as multilevel models. Regarding the most adequate random structure, we followed the top-down strategy suggested by Brady and West [22]: several models with the same full structure (i.e., with all possible variables and interactions) but with a different random structure were estimated using restricted maximum likelihood estimation (REML) and compared using the conditional Akaike criterion information (cAIC), such that the model whose cAIC was lower was selected.

We fitted the linear mixed models for each variable response: both Ca^2+^ rise and the AUC of the calcium signal for SOCE and Ca^2+^ store content or the median of the first 30 s of the recording. Specifically, we evaluated the assumptions of the models, such as the normality and homoscedasticity, through both graphical and test methods, since the non-independence of the observations from the same cluster was controlled, precisely, by the linear mixed models. On the one hand, for the normality assumption of both the Pearson residuals and random effects, we used QQ plots, histograms, Pearson residuals against fitted values plots, Shapiro–Wilks tests, and Jarke Bera tests. On the other hand, we employed Pearson residuals versus fitted values plots and Breush–Pagan and Barlett tests for the homoscedasticity assumption. If any model assumption was violated, different approaches were followed depending on the assumption violated: normality violations were addressed by removing outliers and transforming the response with Box–Cox transformation; heteroscedasticity violations were solved using generalized linear mixed models and different variance–covariance matrix structures and weighting observations. Furthermore, re-sampling methods, such as parametric bootstrapping for mixed models, were performed to evaluate the models and to estimate both the coefficients and their confidence intervals. Notably, the results from the bootstrapping procedures agreed with those from the classic linear mixed models that slightly violated the assumptions. That is why, in the real world, some deviations from assumptions are expected, since, if these deviations are not too large, the inferences extracted from the analysis will be acceptable, which is known as the robustness of validity [23]. For example, the non-normality of the residual distribution results in neither bias nor inefficiency from models; indeed, several authors claim that the violation of normality is not a serious problem as a consequence of the central limit theorem and can even be disregarded when the sample size is large (our experiment consisted of nearly 1000 observations), since the residual distribution will approximate to normality [24,25].

Regarding which experimental conditions were different from each other, all possible pairwise comparisons were made based on combinations between levels for the two explanatory variables considered: treatment and cellular line. At first, the expected value for each experimental condition was estimated from a linear mixed model. Secondly, the differences between each pair of expected values for each experimental condition were estimated; *p*-values were corrected using the Tukey method with a significance level of 0.05; and experimental conditions were grouped according to a pairwise comparison, i.e., if there were no differences between two experimental conditions, they belonged to the same group. In the graphs, this is shown by letters, which indicate the group to which each experimental condition belonged. The last step was based on graph theory and was carried out using the Bron–Kerbosch algorithm to find all maximal cliques.

All data analyses and data collection processes were performed in the R environment [26]; the lmer [27] and nlme [28] packages were used for creating linear mixed models, lmer [27] and boot [29,30] packages for performing bootstrap methods, and multcomp [31] and igraph [32] for pairwise comparison and experimental condition grouping.

### 2.10. Data Analysis: Transcriptomic Experiment Based on Microarrays

First at all, the .CEL files, one per sample, were read with R software (oligo and affy packages). Then, samples were subjected to quality control through different methods, such that those samples which did not pass the quality control were removed from the posterior analysis. At first, the distribution of each sample and the possible presence of any batch effect or the need to normalize was tested through box plots and kernel estimators from non-normalized data. Then, pattern matching was conducted. To achieve this, it was necessary to normalize (quantile) and transform (log_2_) the datasets to make the samples comparable between them. Next, a principal component analysis (PCA) across all samples was conducted, and outlier detection was carried out by determining the robust Mahalanobis distance within each class (group of interest, e.g., observations from NCM460 cells treated with DFMO), where the input variables were the first principal components such that they explained at least 70% (PC70%) of the variability [33]. Another pattern-matching method employed was hierarchical cluster analysis with the Ward method, PC70%, and the Euclidean distance. Finally, probe-level models were employed from which both the normalized unscaled standard error (NUSE) and the relative log expression (RLE) were extracted and evaluated [34].

After quality control, the dataset was preprocessed, i.e., the dataset background was corrected, normalized, and finally summarized, all through the RMA method [35]. Subsequently, data were filtered by employing a non-specific filter implemented by the gene filter R function, and we kept those genes (probes) whose interquartile ranges (across all samples) were larger than the median of all interquartile ranges. Furthermore, we removed all those genes whose ALIAS annotation was unknown. Nevertheless, since we were concerned about a near 80 calcium gene set, all of them were retained in the dataset after the filtering process. In this way, the dataset passed from 138,745 to 12,933 probes. Afterward, differential expression analysis was carried out through linear models, specifically, through linear models for the microarray data (limma) method [36]. Regarding the variables in the model, the fixed factors were cellular line (levels: NCM460 and HT29) and treatment (levels: control and DFMO), whereas the random factor was day, i.e., the day on which the experimental units were processed, taking into account that each day a replicate corresponding to each experimental condition was processed. Then, the linear model selected was:

$$\log_{2}\left(\mathrm{Expression}\right)=\mathrm{Cellular}\;\mathrm{Line}\;+\;\mathrm{Treatment}\;+\;\mathrm{Cellular}\;\mathrm{line}\;\times \;\mathrm{Treatment}\;+\left(\mathrm{Day}\right)$$

where Day is a random factor. Finally, to control I-type error rates, we had to employ the false discovery rate (FDR) [37]. Differences were considered significant at FDR < 0.05.

## 3. Results

### 3.1. Functional Analysis Results

**Figure 3:**
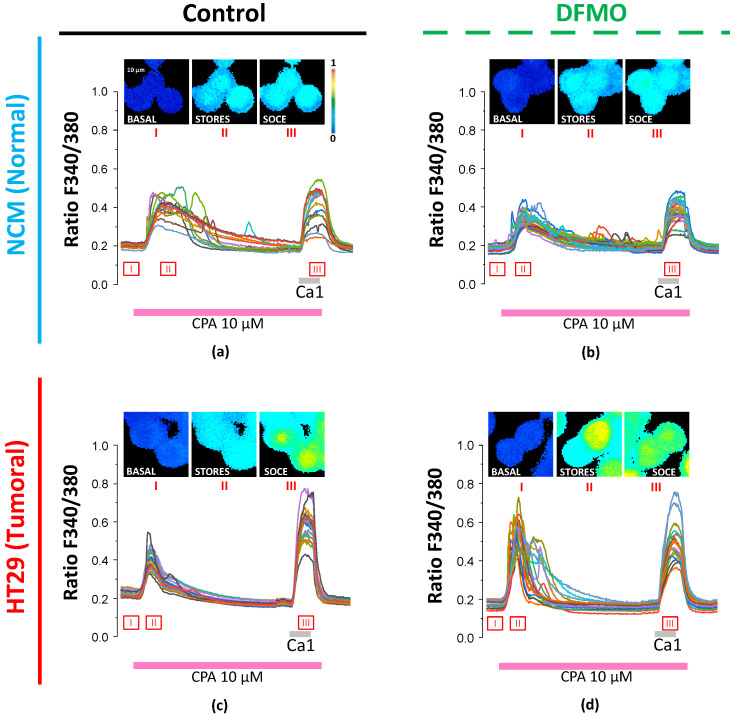
Effects of DFMO treatment on resting Ca^2+^ levels, Ca^2+^ store content, and store-operated Ca^2+^ entry (SOCE) in NCM460 and HT29 cells. Cultures of both normal NCM460 and colon cancer HT29 cells were treated with vehicle (**a**,**c**) or DFMO (**b**,**d**) for 96 h and then loaded with fura2/AM for Ca^2+^ imaging experiments. Resting Ca^2+^ levels (I) were measured before stimulating cells with cyclopiazonic acid (CPA) in Ca^2+^-free media to estimate Ca^2+^ store content (II). Finally, Ca^2+^-containing medium was perfused in the presence of CPA to record SOCE (III). Ca^2+^ images (F340/F380) and single-cell recordings are shown for representative cells located in the same optical field. Images are coded in pseudocolor (color scale showing ratio values between 0 (blue) and 1 (red), corresponding to cytosolic free Ca^2+^ concentration (I), Ca^2+^ store content (II), and SOCE (III)). Data are representative of 8 independent experiments for each condition including 272 NCM460 control cells, 230 HT29 control cells, 303 NCM460 DFMO-treated cells, and 222 HT29 DFMO-treated cells.

**Figure 4:**
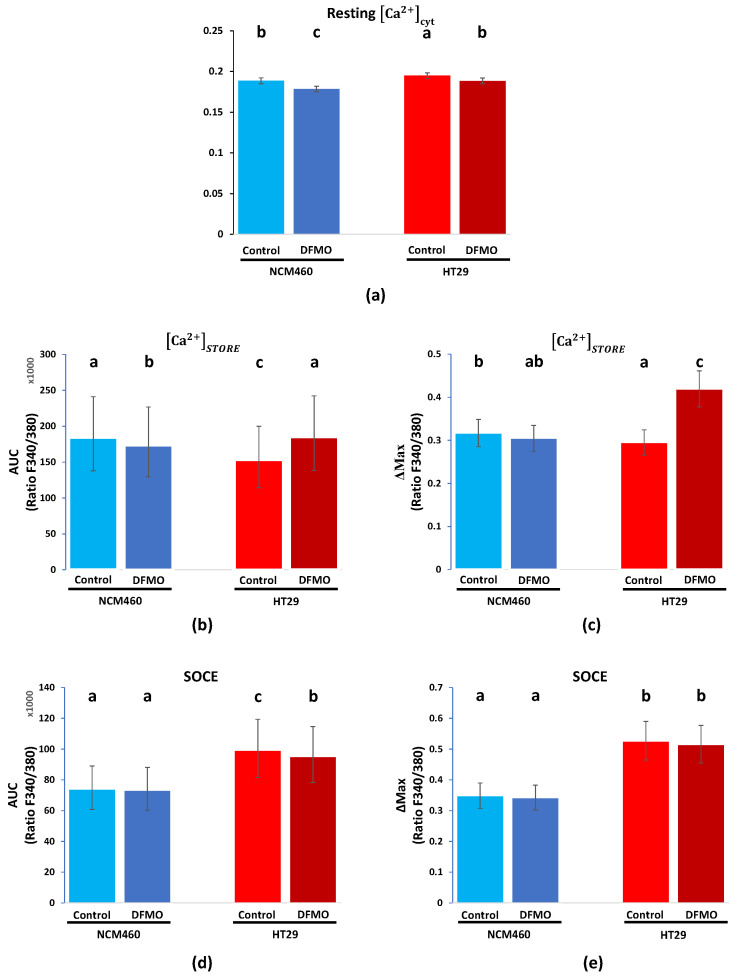
Statistical analysis of the effects of DFMO treatment on resting Ca^2+^ levels (**a**), Ca^2+^ store content (**b**,**c**), and SOCE (**d**,**e**) in NCM460 (blue bars) and HT29 cells (red bars). NCM460 and HT29 cells were pretreated with vehicle (control) or DFMO and subjected to calcium imaging for analysis of resting Ca^2+^ levels, Ca^2+^ store content, and SOCE, as shown in Figure 2. Bar plots represent, in the original units of the response variables (i.e., absolute value, maximum increment, or AUC of F340/380 curves), the expected value (bar) and the SEM (error bars). Experimental conditions were grouped into different clusters according to the adjusted *p* values shown in Table 2, which are indicated by letters: experimental conditions with different letters presented differences; identical letters indicate that the experimental conditions were equal; and those experimental conditions with 2 or more letters (e.g., cluster ab) were similar to those groups with any of these letters (i.e., cluster ab was similar to both cluster a and cluster b). For every experimental condition, 8 replicates were generated, and the total number of cells was: 272 for vehicle-treated NCM460 cells, 230 for vehicle-treated HT29 cells, 303 for DFMO-treated NCM460 cells, and 222 for DFMO-treated HT29 cells.

To assess changes in intracellular Ca^2+^ homeostasis during tumoral transformation (i.e., HT29 vs. NCM460) and the effect of DFMO treatment on each cell line, we employed the calcium imaging technique and Fura2/AM as a fluorescence ratiometric probe. Specifically, we assessed the free resting ${\left[{\mathrm{Ca}}^{2+}\right]}_{\mathrm{cyt}}$, the Ca^2+^ store content, and the SOCE (Figure 3 and Figure 4) in vehicle and DFMO-treated NCM460 and HT29 cells. Firstly, the free basal ${\left[{\mathrm{Ca}}^{2+}\right]}_{\mathrm{cyt}}$ was measured as the median of the absolute F340/F380 signal during the first 30 s of the recordings, and the perfusion medium contained 1 mM Ca^2+^. Secondly, the Ca^2+^ store content was measured as the AUC and Δmax of the signal due to calcium release induced by CPA. Finally, SOCE was assessed as the increase in the AUC and Δmax of the signal due to perfusion with medium containing 1 mM Ca^2+^ after calcium store depletion.

In accordance with our previous results [16], resting Ca^2+^ and SOCE levels appeared higher in colon cancer HT29 cells than in normal NCM460 cells, whereas the Ca^2+^ store content was larger in the normal NCM460 cells than in the colon cancer HT29 cells. Furthermore, DFMO treatment decreased resting [Ca^2+^] and SOCE in colon cancer HT29 cells and increased the Ca^2+^ store content in the same cells. In contrast, DFMO treatment had little or no effect on normal NCM460 cells. These results were similar to our previous results [19].

Figure 4 shows the statistical analysis of these data. Notably, due to the hierarchic structure of the data generated by this kind of experiment, we employed linear mixed models and Box–Cox transformation when needed. Furthermore, through pairwise comparison, the four experimental conditions (i.e., NCM460 control, NCM460 + DFMO, HT29 control, and HT29 + DFMO) were grouped into several clusters, such that the number of conditions in the each group was equal (clusters are indicated by the letters at the top of the bars plotted in Figure 4a–e).

As result, we found that basal ${\left[{\mathrm{Ca}}^{2+}\right]}_{\mathrm{cyt}}$ was significantly higher in non-treated HT29 than in non-treated NCM460 cells, and DFMO treatment decreased it in both cell lines (Figure 4a). Ca^2+^ store content was significantly lower in HT29 control cells than in NCM460 control cells (Figure 4b,c). Furthermore, DFMO treatment affected each cell line differently, since this treatment slightly reduced the calcium store content in NCM460 cells (just in terms of Δmax) but increased it in HT29 cells (in terms of both the AUC and Δmax). Notably, HT29 cells treated with DFMO showed a Ca^2+^ store content equal to that of the NCM460 control in terms of the AUC (Figure 4b,c). Finally, the data (Figure 4d,e) indicated that the SOCE was higher in HT29 cells than in NCM460 cells, and DFMO treatment reduced the SOCE in HT29 cells but not in NCM460 cells. Collectively, these data indicated that DFMO treatment was able to reverse, at least partially, the Ca^2+^ remodeling observed in HT29 cells relative to normal NCM460 cells. Furthermore, our previous results showed that DFMO treatment also reduced proliferation and death resistance in HT29 cells in accordance with the reversal of Ca^2+^ remodeling [19].

We also investigated the effects of DFMO treatment on another colon cancer cell line, SW480. Experiments were carried out again in parallel to the Ca^2+^ imaging experiments in normal NCM460 cells. As expected, the Ca^2+^ store content in colon cancer SW480 cells was much lower than in normal NCM460 cells, whereas the SOCE was higher in SW480 cancer cells than in normal cells. Treatment with DFMO significantly increased the Ca^2+^ stores in SW480 cancer cells but had no effect on normal NCM460 cells. In contrast to HT29 cells, DFMO treatment had no significant effect on the SOCE in SW480 cells (Supplementary Figures S1 and S2).

### 3.2. Transcriptomic Analysis

The transcriptomic differential expression assays that we carried out could be separated into two parts. The first was conducted through two different technologies (RNA-seq and microarrays) to ascertain the differential expression between the HT29 and NCM460 cell lines. Thus, we established the following criteria to consider the information obtained by the two technologies and infer if a gene was differentially expressed or not: a gene was differentially expressed if it was indicated as such by at least one of the two technologies at a 0.05 significance level or by both technologies at a 0.1 significance level, as long as the logFC provided by both technologies fell in the same direction. The second part, i.e., the differential expression analysis between DFMO-treated and non-treated cells, both for HT29 and NCM460 cells, was conducted only by microarrays. Furthermore, the transcriptomic microarray-based experiments were conducted in parallel to the calcium imaging experiment, i.e., each sample assessed through microarrays was extracted from the same culture as a sample assessed by calcium imaging. In addition, both treated and non-treated samples processed on the same day were also extracted from the same culture.

Finally, differential expression analyses were carried out for all genes (65,219 genes with ENSEMBL annotation for RNA-seq and more than 57,500 for Clariom D Human microarray). On the one hand, we identified 16,375 differentially expressed genes (DEGs) between HT29 and NCM460 through Illumina and 12,973 through microarrays. On the other hand, we found out that DFMO treatment was very much selective for HT29 cells, since treatment affected the expression of 3385 genes in HT29 cells against only 61 genes in NCM460 cells. Among all these genes, we focused only on 89 genes related to intracellular Ca^2+^ homeostasis, which we split into six clusters: voltage-operated calcium channels (VOCCs), SOCE modulators, TRP channels, Ca^2+^ release channels, Ca^2+^ pumps and exchangers, and mitochondrial Ca^2+^ transporters. Interestingly, none of these genes were affected in NCM460 cells, as opposed to 17 in HT29 cells. Furthermore, among these 17 genes, the change in the expression of 11 reversed the changes associated with cancer, i.e., the expression of these 11 genes was partially reverted by DFMO treatment. Regarding the effect at the transcriptomic level, DFMO treatment in NCM460 cells affected the expression of only 61 genes, whereas up to 3385 genes were differentially expressed in HT29 cells after treatment, indicating that DFMO is a treatment highly selective for colon cancer cells that is able to reverse, at least partially, the colon cancer phenotype.

**Figure 5:**
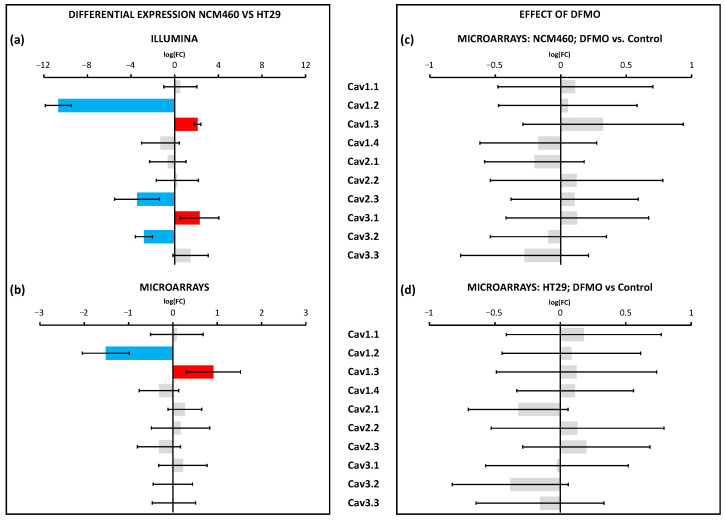
Differential expression of VOCCs between HT29 (colon cancer) and NCM460 (normal colon) cells and effects of DFMO treatment. Bars from bar plots represent $\log_{2}\left(Fold\;Change\right)$, where the error is the confidence interval (*x*-axis), and both of them were estimated by the limma method for several genes (*y*-axis). Grey bars are not statistically different between conditions. Blue bars toward the left indicate significantly lower expression (FDR < 0.05) for cancer cells, whereas red bars toward the right indicate significantly higher expression in cancer cells (FDR < 0.05). (**a**,**b**) Differential expression between cell lines was tested through two different transcriptomic technologies: RNA-seq/Illumina (**a**) and microarrays/Clariom D Human Affymetrix (**b**). (**c**,**d**) Differential expression between cell lines treated and non-treated with DFMO was tested through microarrays/Clariom D Human Affymetrix both for NCM460 (**c**) and HT29 cells (**d**). Fold changes and *p* values are shown in Supplementary Table S1.

**Figure 6:**
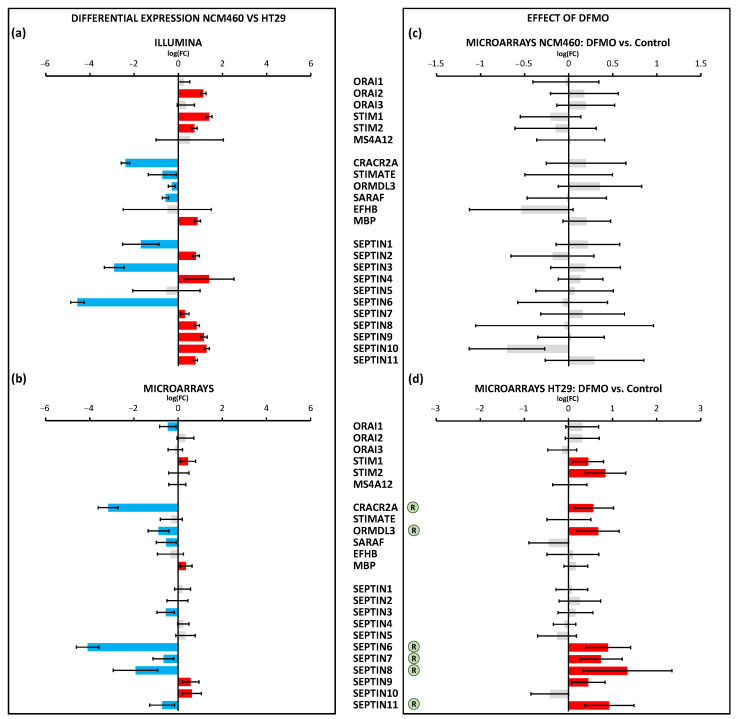
Differential expression between HT29 (colon cancer) and NCM460 (normal colon) cells and between DFMO-treated and non-DFMO-treated cells for those genes that code for members of the store-operated calcium entry (SOCE) mechanism. Bars from bar plots represent $\log_{2}\left(Fold\;Change\right),$ where the errors are the confidence interval (*x*-axis), and both of them were estimated by the limma method for several genes (*y*-axis). Grey bars are not statistically different between conditions. Blue bars toward the left indicate significantly lower expression (FDR < 0.05) for tumoral cells in relation to normal cells, whereas red bars toward the right indicate higher expression (FDR < 0.05). (**a**,**b**) Differential expression between cell lines was tested through two different transcriptomic technologies: RNA-seq/Illumina (**a**) and microarrays/Clariom D Human Affymetrix (**b**). (**c**,**d**) Differential expression between cell lines treated and non-treated with DFMO was tested through microarrays/Clariom D Human Affymetrix for both NCM460 (**c**) and HT29 cells (**d**). The letter “R” in a circle beside the name of a gene means that the differential expression of that gene due to tumoral transformation was reversed after DFMO treatment (there was significant differential expression in terms of $\log_{2}\left(Fold\;Change\right)$, but in the opposite direction to that observed in tumor cells with respect to normal cells). Fold changes and *p* values are shown in Supplementary Table S2.

Regarding VOCCs, our results showed that *CAV1.2* was downregulated in colon cancer HT29 cells, whereas *CAV1.3* was overexpressed in colon cancer HT29 cells (Figure 5a,b). Furthermore, *CAV2.3* and *CAV3.2* were downregulated, and *CAV3.1* and *CAV3.3* were overexpressed according to RNA-seq technology. Regarding the effect of DFMO, no gene belonging to the VOCCs was affected by this treatment (Figure 5c,d). Regarding the 23 genes involved in SOCE, as many as 18 of them were identified as differentially expressed in cancer cells relative to normal cells by RNA-seq technology, whereas only 13 of them were differentially expressed according to microarrays (Figure 6a,b). Specifically, RNA-seq showed the overexpression of *ORAI1*, *STIM1*, *STIM2*, *MBP*, and *SEPTIN2*,*4*,*7-11* and the downregulation of *CRACR2A*, *STIMATE*, *ORMDL3*, *SARAF*, *SEPTIN1*,*3*, and *SEPTIN6* in HT29 cells relative to NCM460 cells (Figure 6a). Microarrays showed the overexpression of *STIM1*, *MBP*, *SEPTIN9*, and *SEPTIN10* and the downregulation of *ORAI1*, *CRACR2A*, *ORMDL3*, *SARAF*, *SEPTIN3*,*6-8*, and *SEPTIN11* (Figure 6b). Accordingly, both technologies reported the overexpression of *STIM1*, *MBP*, *SEPTIN9*, and *SEPTIN10* and the downregulation of *CRACR2A*, *ORMDL3*, *SARAF*, *SEPTIN3*, and *SEPTIN6* in colon cancer cells. Thus, some discrepancies occurred for *SEPTIN7*,*8* and *SEPTIN11*, so these genes were not considered as differentially expressed. Consequently, the data suggested that HT29 cells overexpressed *ORAI2*, *STIM1*, *STIM2*, *MBP*, *SEPTIN2*, *SEPTIN4*, *SEPTIN9*, and *SEPTIN10* relative to NCM460 cells, and showed a lower expression of *ORAI1*, *CRACR2A*, *STIMATE*, *ORMD3*, *SARAF*, *SEPTIN1*, *SEPTIN3*, and *SEPTIN6* relative to the normal cells. Notably, treatment with DFMO did not affect NCM460 but did affect HT29 cells (Figure 6c,d). Specifically, DFMO treatment induced the overexpression of *STIM1*, *STIM2*, *CRACR2A*, *ORMDL3*, *SEPTIN6-9*, and *SEPTIN11* in HT29 cells only (Figure 6d). Thus, it was clear that DFMO treatment was able to partially reverse the differential expression found for *CRACR2A*, *ORMDL3*, and *SEPTIN6*.

Furthermore, if we consider the differential expression between HT29 and NCM460 cells identified by microarrays, DFMO treatment was also able to partially reverse the differential expression of *SETPIN7*, *SEPTIN8*, and *SEPTIN11*.

**Figure 7:**
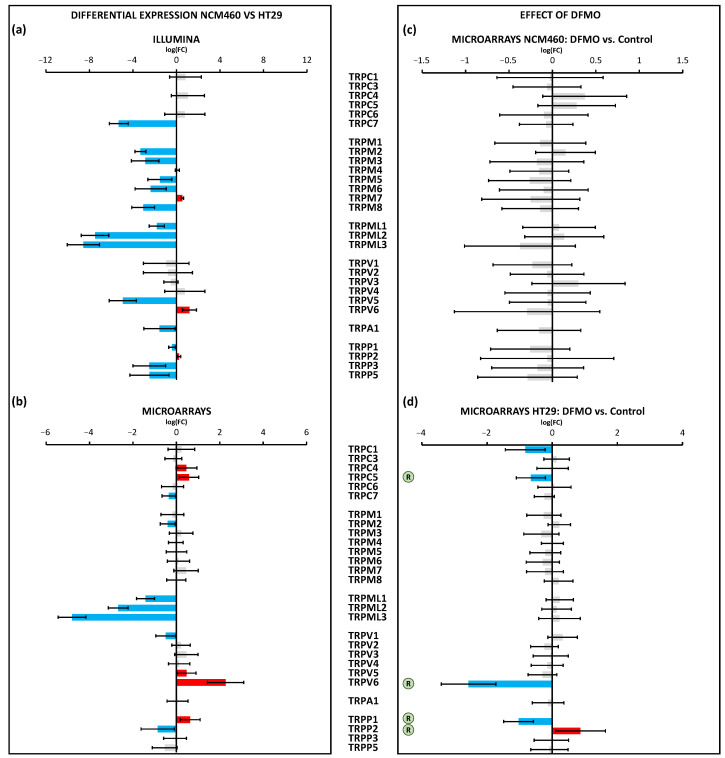
Differential expression of TRP channel genes between HT29 (colon cancer) and NCM460 (normal colon) cells and between DFMO-treated and non-DFMO-treated cells. Bars from bar plots represent $\log_{2}\left(Fold\;Change\right)$, where the errors are the confidence interval (*x*-axis), and both of them were estimated by the limma method for several genes (*y*-axis). Grey bars are not statistically different between conditions. Blue bars toward the left indicate significantly lower expression (FDR < 0.05) for tumoral cells in relation to normal cells, whereas red bars toward the right indicate higher expression (FDR < 0.05). (**a**,**b**) Differential expression between cell lines was tested through two different transcriptomic technologies: RNA-seq/Illumina (**a**) and microarrays/Clariom D Human Affymetrix (**b**). (**c**,**d**) Differential expression between cell lines treated and non-treated with DFMO was tested through microarrays/Clariom D Human Affymetrix for both NCM460 (**c**) and HT29 cells (**d**). The letter “R” in a circle beside the name of a gene means that the differential expression of that gene due to tumoral transformation was reversed after DFMO treatment (there was significant differential expression in terms of $\log_{2}\left(Fold\;Change\right)$, but in the opposite direction to that observed in tumor cells with respect to normal cells). Fold changes and *p* values are shown in Supplementary Table S3.

Regarding the 27 genes that coded for TRPs, the most remarkable result was that over half of them were downregulated in HT29 cells relative to NCM460 cells (Figure 7a,b). Specifically, by combining the outcomes from both transcriptomic technologies, we could infer, on the one hand, that the following 13 genes were less expressed in HT29 cells: *TRPC7*, *TRPM2*, *TRPM3*, *TRPM5*, *TRPM6*, *TRPM8*, *TRPML1-3*, *TRPV1*, *TRPA1*, *TRPP3*, and *TRPP5* (Figure 7c,d). On the other hand, it was clear that *TRPC4*, *TRPC5*, and *TRPV6* were upregulated in HT29 cells. It is important to note that three genes showed an opposite differential expression pattern according to both technologies: *TRPV5, TRPP1*, and *TRPP2*. Regarding the effects of DFMO treatment, again, the treatment affected HT29 but not NCM460 cells (Figure 7c,d). Specifically, DFMO treatment decreased the expression of *TRPC1*, *TRPC5*, *TRPV6*, and *TRPP1*, whereas it increased the expression of *TRPP2* (Figure 7d). Thus, DFMO treatment was able to partially reverse the tumoral phenotype in terms of the expression of *TRPC5* and *TRPV6*, and, by taking into account just the results from the microarrays, DFMO treatment also partially reversed the differential expression of both *TRPP1* and *TRPP2*.

**Figure 8:**
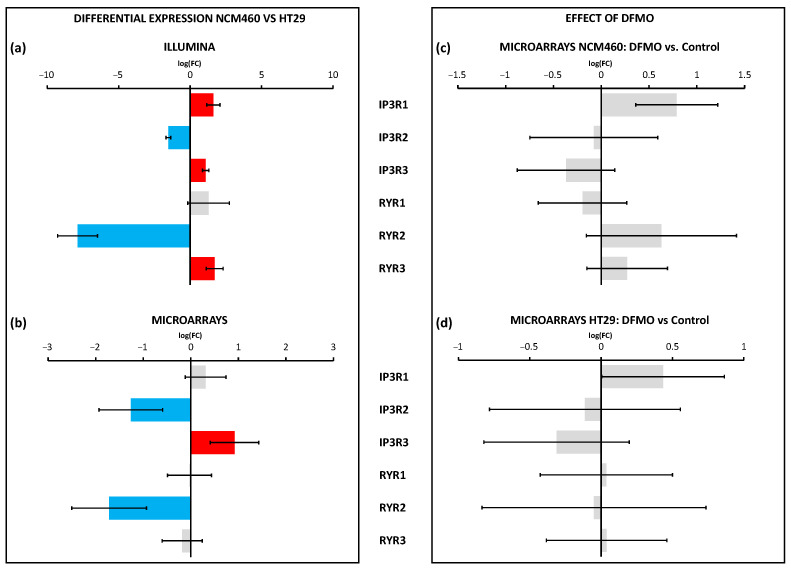
Differential expression of Ca^2+^ release channel genes between HT29 (colon cancer) and NCM460 (normal colon) cells and between DFMO-treated and non-DFMO-treated cells. Bars from bar plots represent $\log_{2}\left(Fold\;Change\right)$, where the errors are the confidence interval (*x*-axis), and both of them were estimated by the limma method for several genes (*y*-axis). Grey bars are not statistically different between conditions. Blue bars toward the left indicate significantly lower expression (FDR < 0.05) for tumoral cells in relation to normal cells, whereas red bars toward the right indicate higher expression (FDR < 0.05). (**a**,**b**) Differential expression between cell lines was tested through two different transcriptomic technologies: RNA-seq/Illumina (**a**) and microarrays/Clariom D Human Affymetrix (**b**). (**c**,**d**) Differential expression between cell lines treated and non-treated with DFMO was tested through microarrays/Clariom D Human Affymetrix for both NCM460 (**c**) and HT29 cells (**d**). Fold changes and p values are shown in Supplementary Table S4.

Regarding the expression of genes coding for Ca^2+^ release channels, our results indicated that Illumina identified five out of six genes belonging to the CRC set as differentially expressed between HT29 and NCM460 cells, against only three identified by microarrays (Figure 8a,b). Combining the results from both technologies, our findings suggested that *IP3R1* and *IP3R3* were upregulated in HT29 cells, whereas *IP3R2* and *RYR2* were downregulated. Nevertheless, *RYR3* may also be overexpressed in HT29 cells. However, DFMO treatment had no effect on gene expression either in NCM460 or HT29 cells, suggesting that the possible differences could be mediated by excess polyamines.

**Figure 9:**
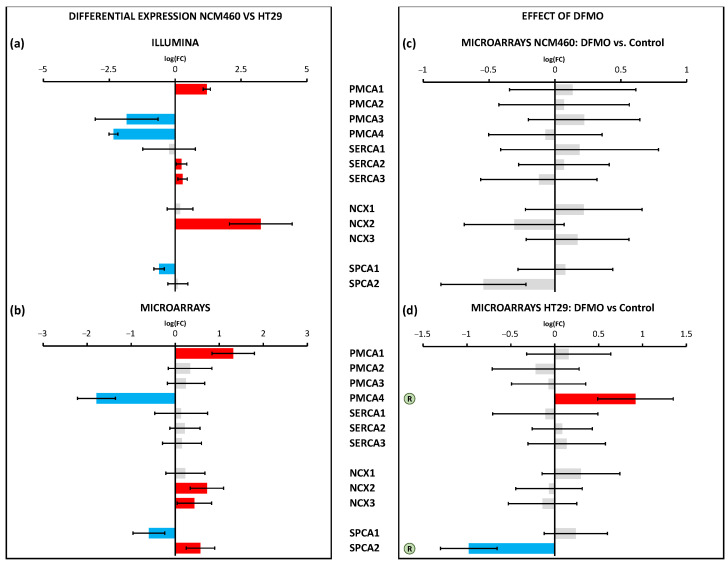
Differential expression of Ca^2+^ extrusion genes between HT29 (colon cancer) and NCM460 (normal colon) cells and between DFMO-treated and non-DFMO-treated cells. Bars from bar plots represent $\log_{2}\left(Fold\;Change\right)$, where the errors are the confidence interval (*x*-axis), and both of them were estimated by the limma method for several genes (*y*-axis). Grey bars are not statistically different between conditions. Blue bars toward the left indicate significantly lower expression (FDR < 0.05) for tumoral cells in relation to normal cells, whereas red bars toward the right indicate higher expression (FDR < 0.05). (**a**,**b**) Differential expression between cell lines was tested through two different transcriptomic technologies: RNA-seq/Illumina (**a**) and microarrays/Clariom D Human Affymetrix (**b**). (**c**,**d**) Differential expression between cell lines treated and non-treated with DFMO was tested through microarrays/Clariom D Human Affymetrix for both NCM460 (**c**) and HT29 cells (**d**). The letter “R” in a circle beside the name of a gene means that the differential expression of that gene due to tumoral transformation was reversed after DFMO treatment (there was significant differential expression in terms of $\log_{2}\left(Fold\;Change\right)$, but in the opposite direction to that observed in tumor cells with respect to normal cells). Fold changes and p values are shown in Supplementary Table S5.

The calcium extrusion systems were also differentially expressed between HT29 and NCM460 cells (Figure 9a,b). Indeed, among the 12 genes considered, seven were identified as DEGs by Illumina (*PMCA1*, *SERCA2*,*3*, and NCX2 overexpressed, and *PMCA*3,*4* and *SPCA1* less expressed in HT29) and six by microarrays (*PMCA1*, *NCX2*,*3*, and *SPCA2* overexpressed, and *PMCA4* and *SPCA1* less expressed in HT29). Again, DFMO treatment only affected HT29 cells and not NCM460 cells. Interestingly, tumor cells showed an increased expression of *PMCA4* and decreased expression of *SPCA2*, and DFMO treatment reversed both changes in HT29 cells (Figure 9c,d).

**Figure 10:**
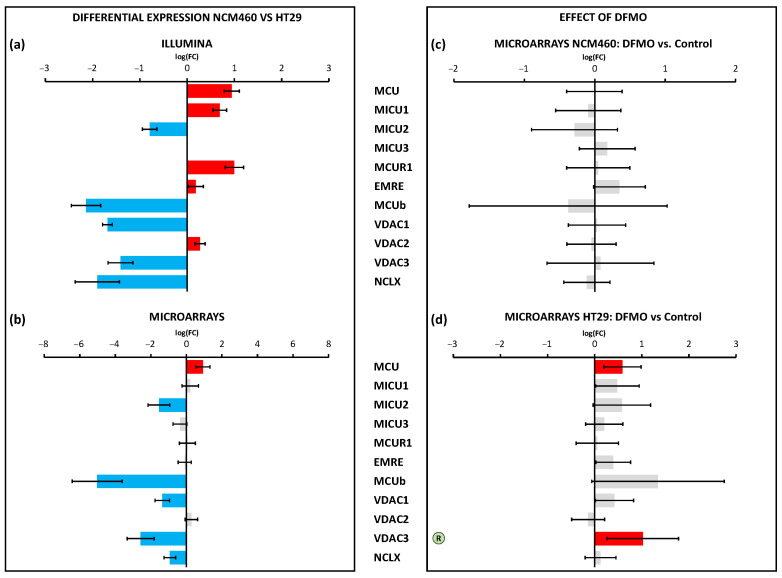
Differential expression of genes coding for mitochondrial Ca^2+^ transport between HT29 (colon cancer) and NCM460 (normal colon) cells and between DFMO-treated and non-DFMO-treated cells. Bars from bar plots represent $\log_{2}\left(Fold\;Change\right)$, where the errors are the confidence interval (*x*-axis), and both of them were estimated by the limma method for several genes (*y*-axis). Grey bars are not statistically different between conditions. Blue bars toward the left indicate significantly lower expression (FDR < 0.05) for tumoral cells in relation to normal cells, whereas red bars toward the right indicate higher expression (FDR < 0.05). (**a**,**b**) Differential expression between cell lines was tested through two different transcriptomic technologies: RNA-seq/Illumina (**a**) and microarrays/Clariom D Human Affymetrix (**b**). (**c**,**d**) Differential expression between cell lines treated and non-treated with DFMO was tested through microarrays/Clariom D Human Affymetrix for both NCM460 (**c**) and HT29 cells (**d**). The letter “R” in a circle beside the name of a gene means that the differential expression of that gene due to tumoral transformation was reversed after DFMO treatment (there was significant differential expression in terms of $\log_{2}\left(Fold\;Change\right)$, but in the opposite direction to that observed in tumor cells with respect to normal cells). Fold changes and p values are shown in Supplementary Table S6.

We also studied the differential expression of genes involved in mitochondrial Ca^2+^ transport. These genes showed clear differential expression between both cell lines (Figure 10a,b). On the one hand, Illumina identified five overexpressed genes (*MCU*, *MICU1*, *MCUR1*, *EMRE*, and *VDAC2*) and five downregulated genes (*MICU2*, *MCUb*, *VDAC1*, *VDAC3*, and *NCLX*) in HT29 cells. On the other hand, microarrays identified just one overexpressed gene, MCU (also identified by Illumina), and the same five downregulated genes identified by Illumina. Again, DFMO treatment affected only HT29 cells (Figure 10c,d). Specifically, DFMO treatment upregulated the expression of *MCU* and *VDAC3*, indeed reversing the effects of cancer in the case of VDAC3. Western blotting confirmed that MCU was upregulated by DFMO treatment in HT29 cells at the protein level (Supplementary Figure S3).

**Figure 11:**
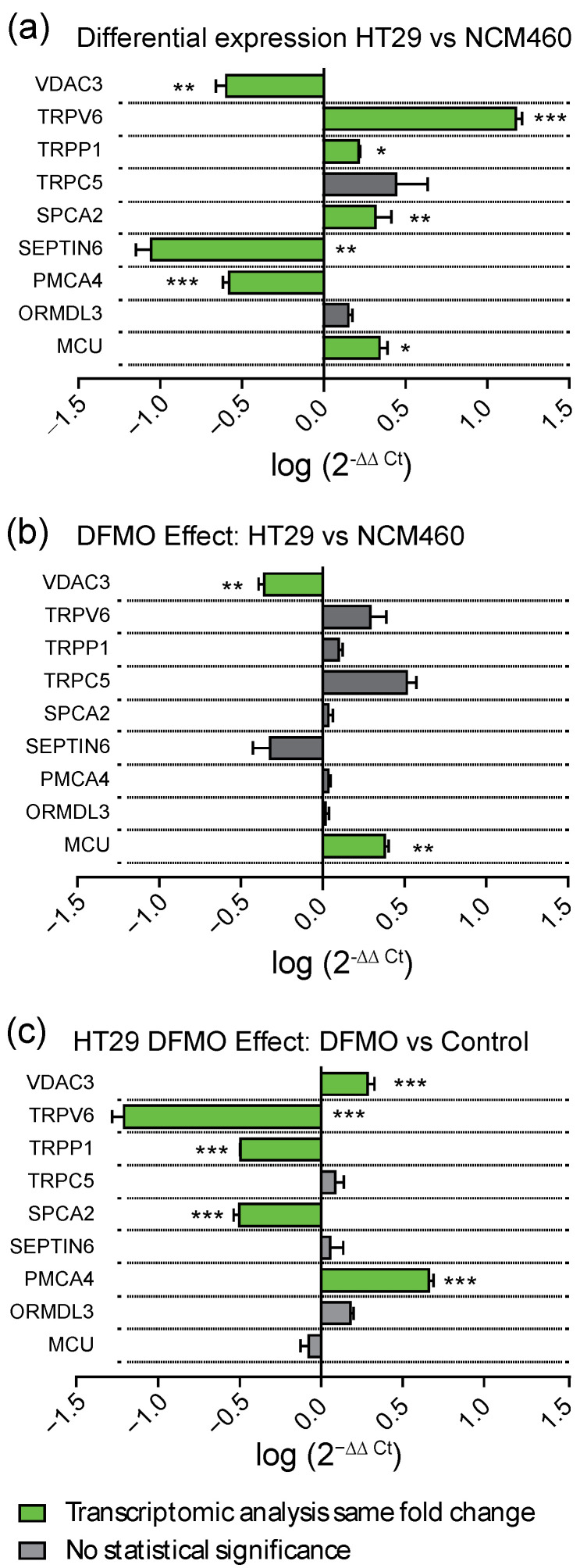
qPCR analysis of differential expression of selected genes in HT29 and NCM460 cells in control and DFMO-treated cells. (**a**) mRNA expression levels in cancer cells (HT29) were calculated using control cell (NCM460) expression levels as calibrator, and the ribosomal RP18S gene was used as housekeeping gene. In this representation, a value of 0 indicates no change, negative values indicate decreased expression, and positive values indicate increased expression in HT29 cells relative to NCM460. (**b**) mRNA levels of those genes coding selected Ca^2+^ transport system proteins in DFMO-treated HT29 cells relative to DFMO-treated NCM460 cells. (**c**) Differential expression of selected genes between DFMO-treated and non-treated HT29 cancer cells. Each data point was obtained from triplicate determinations from at least three different samples. Data were analyzed using the threshold cycle (Ct) relative quantification method (ΔΔCt), and the relative abundance of the genes was calculated from 2(^−ΔCt^). Bar plots represent log(2^−∆∆Ct^) ± SEM. Statistical analysis in this plot was conducted by Student’s t test with Bonferroni´s correction for two independent samples (ΔCt(HT29) and ΔCt(NCM460)) obtained with RP18S as endogenous control. For the sake of clarity, statistically significant changes are plotted in green. * *p* < 0.05, ** *p* < 0.01, *** *p* < 0.001.

In order to validate the most important results, the differential expression of selected genes was confirmed by qPCR analysis. For this analysis, we selected those genes whose differential expression in cancer cells vs. normal cells was reversed by DFMO treatment. The selected genes included those coding for channels TRPV6, TRPP1, and TRPC5; the SOCE modulators SEPTIN6 and ORMDL3; the pumps SPCA2 and PMCA4; and the mitochondrial transporters VDAC3 and MCU. Figure 11a confirms that all selected gene transcripts were differentially expressed in HT29 colon cancer cells relative to normal NCM460 cells, except for TRPC5 and ORMDL3, which showed no significant difference. Interestingly, when we compared the expression of the same selected genes in DFMO-treated normal and tumor cells, most differential expression was largely dampened (Figure 11b), thus also confirming the microarray data shown above. Finally, when we compared qPCR data on the expression of the selected genes before and after DFMO treatment in HT29 cells, the results confirmed that, in accordance with the microarray data shown above, DFMO treatment significantly increased the expression of the Ca^2+^ extrusion systems PMCA4 and VDAC3 in HT29 colon cancer cells and decreased the expression of the TRPV6 and TRPP1 channels and the Orai1 positive modulator SPCA2 (Figure 11c). However, in contrast to the microarray data, the qPCR data did not confirm the effects of DFMO treatment on TRPC5, SEPTIN6, ORMDL3, or MCU (Figure 11c).

## 4. Discussion

We investigated whether polyamine synthesis inhibition using DFMO as an ODC suicide inhibitor was able to reverse Ca^2+^ remodeling in cancer cells. To this end, HT29 and NCM460 cells were used as well-established cell models representing CRC and normal colonic cells, respectively [16]. CRC cells displayed significantly enhanced levels of resting intracellular [Ca^2+^] and SOCE relative to normal cells. Likewise, CRC cells showed decreased Ca^2+^ store content relative to normal cells. These findings are similar to previous results reported in [16,17]. We also showed here that SW480 colon cancer cells displayed enhanced SOCE and decreased Ca^2+^ stores compared to NCM460 cells, thus resembling the same intracellular Ca^2+^ remodeling as HT29 colon cancer cells. Although these changes were observed in cell lines representative of normal and tumor cells and should be confirmed in primary normal and tumor cells, changes in intracellular [Ca^2+^] homeostasis, collectively referred to as Ca^2+^ remodeling, could be part of the phenotypic changes associated with carcinogenesis; they may contribute to the hallmarks of cancer displayed by CRC cells, including enhanced cell proliferation and resistance to cell death [18]. We found that the incubation of cells with DFMO, a treatment that abolishes polyamine biosynthesis and decreases levels of intracellular polyamines, also significantly decreased both resting Ca^2+^ and SOCE, whereas it increased the Ca^2+^ store content in CRC cells, thus reversing the hypothetical Ca^2+^ remodeling associated with CRC. These results are similar to our recently reported results [19]. In contrast, in normal cells, the effects of DFMO were less important. These data suggested that polyamine synthesis inhibition may reverse, at least partially, the hypothetical Ca^2+^ remodeling associated with CRC, having minor effects on normal cells, where the ODC expression is low. Conversely, our results suggested that the excess polyamine synthesis observed in c-myc-related cancers may contribute to the hypothetical Ca^2+^ remodeling associated with CRC.

The transcriptomic analysis of normal and CRC cells treated with DFMO showed that polyamine synthesis inhibition was able to significantly modify a large fraction (nearly 25%) of all gene transcripts studied in CRC cells. In striking contrast, in normal colonic cells, the same treatment affected less than 0.5% of the transcripts. Therefore, DFMO treatment was highly specific for cancer cells and had a nearly negligible effect on normal colonic cells. Again, we could explain these results by the fact that normal cells do not express ODC or express very low ODC levels unless this gene is induced during epithelial restitution, for instance. In this scenario, DFMO treatment probably induces only off-target effects or nearly negligible effects due to polyamine synthesis inhibition. These data also suggested that polyamines may induce rather dramatic effects on the transcriptome and the differential expression of nearly 25% of all the transcripts studied.

**Figure 12:**
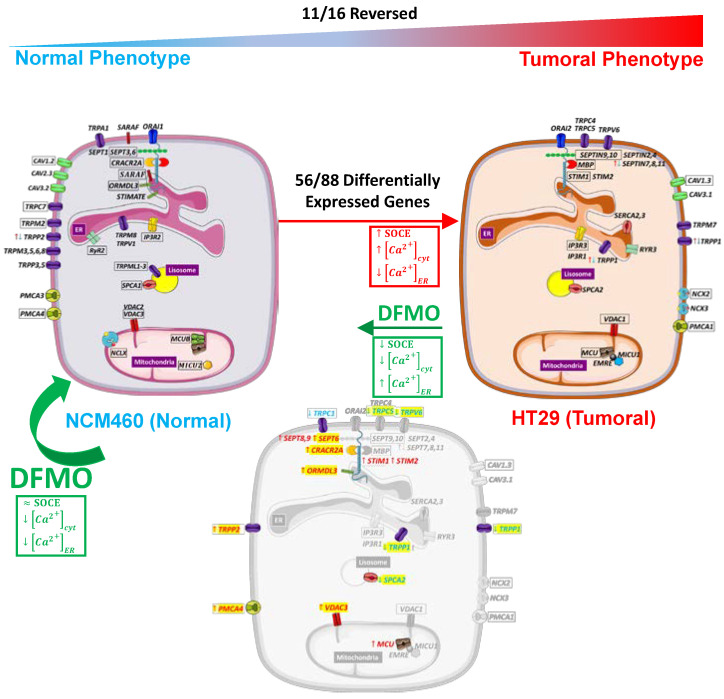
Molecular basis for the hypothetical remodeling of intracellular Ca^2+^ homeostasis in cancer and its reversal by polyamine synthesis inhibition. At the top of figure is displayed the differential expression between HT29 (**right**) and NCM460 (**left**) cell lines for only those genes of interest (i.e., genes coding for Ca^2+^ transport systems or modulators), which showed differential expression at a 0.05 signification level. Specifically, in the HT29 cell are displayed the genes that were upregulated in HT29 cells relative to NCM460 cells. The NCM460 cell shows genes that were upregulated in normal cells. Those genes whose differential expression was equal when assessed by both microarray and RNA-seq methods are surrounded by a black frame. Genes whose differential expression differed between the methods are indicated by two up and down arrows. Boxes next to each cell indicate the expected functional changes due to remodeling and its reversal (DFMO treatment). At the bottom of figure, indicated by a green arrow to the left, are displayed the changes observed in HT29 cells after DFMO treatment. HT29 cells treated with DFMO are halfway between HT29 and NCM460 cells. Genes which were differentially expressed after DFMO treatment include upregulated genes in red and downregulated genes in blue. Genes whose differential expression was reversed by DFMO treatment are highlighted in yellow. The effects of DFMO on the expression of TRPV6, TRPP1, SPCA2, PMCA4, and VDAC3 were confirmed by qPCR analysis.

In this work, we focused on the transcriptional effects on genes directly involved in intracellular Ca^2+^ homeostasis. We analyzed the effects of DFMO treatment (polyamine synthesis inhibition) on the transcription of the 10 voltage-gated Ca^2+^ channels, the 23 genes known to be involved in SOCE, the 28 TRP channels, the 6 Ca^2+^ release channels of the ER, the 12 Ca^2+^ pumps and exchangers, and the 11 genes known to be involved in mitochondrial Ca^2+^ transport. Figure 12 summarizes the changes in the Ca^2+^ transport systems in cancer cells relative to normal cells and the effects of DFMO. We used two independent approaches for transcriptomic analysis, i.e., Illumina next-generation RNA sequencing (RNAseq) and microarrays. Illumina data include sequence data that are critical in cases where polymorphisms or mutations are the main target. Microarrays only provide data on the relative transcription level of selected sequences. We selected microarrays instead of RNAseq for our analysis of the effects of DFMO treatment for several reasons. First, because microarray sensitivity is lower than that of RNAseq, we expected that the differentially expressed genes would be those more significantly influenced by DFMO treatment, leading to less artefactual DEGs. Second, the computational and preprocessing methods of transcriptomic outcomes are simpler with microarrays, i.e., RNAseq outcomes warrant more sophisticated and more computationally powerful methods than microarray outcomes. Finally, transcriptomic microarrays are generally more affordable.

Regarding voltage-gated Ca^2+^ channels, the data obtained using Illumina were quite similar but not identical to our previous results obtained using Ion Torrent technology [18]. Microarray analysis showed that only two transcripts were differentially expressed in cancer cells, whereas changes in three other transcripts were only significant according to Illumina and not microarray. Specifically, we found that Cav1.2 was downregulated, whereas Cav1.3 was upregulated in CRC cells. Polyamine synthesis inhibition had no effect on the expression of any of the voltage-gated Ca^2+^ channels, either in the CRC cells or in the normal cells. Accordingly, these data indicated that, although the previous results suggested the differential expression of certain voltage-gated Ca^2+^ channels in CRC, they could not be attributed to excess polyamine synthesis in CRC.

In marked contrast, a number of molecular players involved in SOCE (ORAI1,2,3 and STIM1,2) and its modulatory proteins were differentially expressed in CRC cells relative to normal cells. Moreover, the transcription levels of SOCE molecular players were deeply influenced by polyamine synthesis inhibition. However, these changes were only observed in CRC and not in normal colon cells. Specifically, we found that DFMO treatment induced the increased expression of Stim1 and Stim2 transcripts in HT29 cells. These data are similar to our previous results using qRT-PCR [19]. However, at the protein level, DFMO treatment decreased Stim1 expression in HT29 cells without changing Stim2 expression [19]. In addition, DFMO treatment increased the expression of a number of SOCE modulators, including CRACR2A, ORMDL3, and several septins. In nearly all these cases, except for SEPTIN9, the change induced by DFMO reversed the change associated with cancer. These data suggested that a number of SOCE modulators are downregulated in CRC, leading to a loss in SOCE modulation that might contribute to the enhanced SOCE in cancer cells. However, in contrast to the microarray data, the qPCR data did not confirm that DFMO increased either SEPTIN6 or ORMDL3. Although qPCR is considered the gold-standard for testing differential expression, the larger number of primers used for the microarrays (20 instead of just one for qPCR) may explain this difference. Further research is required, therefore, to confirm the effects of the polyamine depletion of SOCE modulators.

SPCA2, a secretory pathway Ca^2+^ ATPase that has recently been reported to interact and modulate Orai1 channels to activate them independently of store depletion [38], was overexpressed in CRC cells. Interestingly, we found that SPCA2 was downregulated after DFMO treatment in HT29 colon cancer cells but not in normal cells. These data were also confirmed by qPCR analysis. Therefore, these data suggested that overexpressed SPCA2 in CRC cells might contribute to enhance resting [Ca^2+^] and Orai1 activation independently of store depletion in CRC, a process that is limited by polyamine synthesis inhibition. In addition, it has recently been reported that the epigenetic modulation of SPCA2 reverses the epithelial-to-mesenchymal transition in breast cancer cells [39]. It is tempting to speculate that overexpressed SPCLA2 in CRC cells due to polyamine excess may play a similar, previously unrecognized role in CRC as well.

TRP channels were also differentially expressed in CRC cells relative to normal cells. In general, both the Illumina and microarray technologies showed the decreased expression of a number of different TRP channels in CRC cells, particularly TRPML1,2,3 and TRPP2. However, CRC cells also showed the enhanced expression of a few TRP channels, including the TRPC5, TRPV6, and TRPP1 channels, that may have contributed to the appearance of non-selective store-operated currents characteristic of CRC cells [16]. qPCR data confirmed these results for TRPV6 and TRPP1 but not for TRPC5. This finding for TRPC5 could have been due to the extremely low number of copies of TRPC5 transcripts. Again, polyamine synthesis inhibition had no effect on the transcription of TRP channels in normal cells. In contrast, in CRC cells, polyamine synthesis inhibition decreased the expression of the TRPC1 and 5, TRPV6, and TRPP1 channels, whereas it increased TRPP2 gene transcription. The pPCR data also confirmed the effects of DFMO on the expression of TRPV6 and TRPP1. We reported previously that DFMO treatment decreased the expression of TRPC1 in HT29 cells at the protein level [19]. Further research is required to validate these additional changes in the TRP channel expression at the protein level.

The data on TRP channel remodeling induced by DFMO treatment strongly suggested that polyamines may influence cancer hallmarks acting on the transcriptional level of these TRP channels. For instance, TRPV6, the epithelial Ca^2+^ channel modulated by the vitamin D receptor, has also been previously related to CRC and other forms of cancer [40]. The cases of TRPP1 and TRPP2 are intriguing, as these are channel complexes involved in sensing shear stress in epithelia that are present in the ER. The results indicated an increased TRPP1/TRPP2 ratio in CRC cells, a feature that was also reversed by polyamine synthesis inhibition. Further research is required to understand the role of the TRPP1 and 2 channels in colon cancer and their relation to polyamine synthesis.

The Ca^2+^ release channels of the ER, including IP_3_ and ryanodine receptors, were differentially expressed in cancer cells and may also contribute to cancer hallmarks. However, none of these Ca^2+^ release channels were modulated by polyamine synthesis inhibition, either in normal or CRC cells, at least at the transcriptional level, thus excluding the possibility that changes in the transcription of these channels are mediated by ODC overexpression. Interestingly, as mentioned above, the TRPP1 and TRPP2 channels can also be found at the ER membranes, where they could play a role as leak channels. Specifically, TRPP2 is a calcium-permeant transient receptor potential (TRP) cation channel expressed primarily on the ER membrane and primary cilia of all cell and tissue types [41]. TRPP2 mutations lead to autosomal-dominant polycystic kidney disease. Recent data indicate that TRPP2 is involved in susceptibility to cell death induced by stress [41]. Accordingly, changes in the TRPP1/TRPP2 ratio related to CRC and polyamine synthesis could be involved in Ca^2+^ store content, Ca^2+^ transfer from the ER to mitochondria, and sensitivity to stress. Again, further research is required to validate this possibility.

Regarding Ca^2+^ extrusion systems such as Ca^2+^ pumps and transporters, the data showed the differential expression of several transport systems, including the enhanced expression of the pumps PMCA1 and SPCA2 and the exchanger NCX2, along with the decreased transcription of the pumps PMCA4 and SPCA1. Again, polyamine synthesis inhibition had no effect on the transcription of any of these calcium extrusion systems in normal cells. However, in CRC cells, polyamine synthesis inhibition reversed the changes in the transcription of PMCA4 and SPCA2 linked to CRC. These results were confirmed by qPCR analysis. As stated above, SPCA2 may interact and modulate Orai1 to activate Orai1 independently of both Stim1 and Ca^2+^ store depletion, thus leading to store-independent Ca^2+^ entry. PMCA4, the plasma membrane Ca^2+^ ATPase linked previously to CRC [42], was downregulated in CRC, likely contributing to enhanced resting intracellular [Ca^2+^]. This gene returned to normal levels after DFMO treatment, thus probably contributing to decreased resting Ca^2+^ levels in treated cells.

Finally, mitochondrial Ca^2+^ transport systems were differentially expressed in CRC cells. Most changes involved the decreased expression of the molecular players involved in the control of the mitochondrial Ca^2+^ uniporter (MCU) and the Ca^2+^ channel involved in mitochondrial Ca^2+^ uptake. Once more, polyamine synthesis inhibition had no effect on the transcription levels of the mitochondrial Ca^2+^ transport systems in normal cells. However, in cancer cells, polyamine synthesis inhibition increased the expression of both MCU and VDAC3. qPCR analysis also confirmed the increased expression of VDAC3 in DFMO-treated HT29 cells. We used western blotting to confirm that MCU expression was also enhanced at the protein level in HT29 cells treated with DFMO (Supplementary Figure S3). Therefore, the modulation of the transcription of these two particularly relevant mitochondrial channels in CRC could be mediated by ODC and polyamines. Interestingly, these changes may contribute to explaining the reversal of the cancer hallmarks related to susceptibility to apoptosis. In other words, the enhanced resistance to apoptosis characteristic of CRC cells could be mediated, at least partially, by changes in the expression of MCU and VDAC3 due to ODC activation.

In summary, our results indicated that a few genes involved in Ca^2+^ transport appeared to be specifically modulated by polyamines in CRC cells and could be responsible for most changes in intracellular Ca^2+^ homeostasis in CRC cells. Thus, an excess of polyamines could induce the downregulation of SOCE modulatory genes (STIM1,2), the plasma membrane Ca^2+^ ATPase PMCA4 Ca^2+^ pump, and the mitochondrial Ca^2+^ channels MCU and VDAC3, contributing to dysregulated SOCE and enhanced resting Ca^2+^ and mitochondrial Ca^2+^ uptake in CRC. Conversely, polyamines could also induce the overexpression of TRPV6 channels, the Orai1 activator SPCA2, and the exchange of TRPP1 channels by TRPP2, thus likely contributing to enhanced Ca^2+^ entry and decreased Ca^2+^ store content in CRC. These findings may provide new insights into the role of polyamines in Ca^2+^ remodeling in cancer. Further research in animal models and/or tumor samples from patients is required to validate our findings.

## 5. Conclusions

We conclude that polyamine synthesis inhibition may partially reverse changes in intracellular calcium homeostasis hypothetically associated with CRC, including a decrease in resting intracellular Ca^2+^ and store-operated Ca^2+^ entry, as well as an increase in Ca^2+^ store content. These effects were observed in CRC cells but not in normal colonic cells. Analogously, polyamine synthesis inhibition induced the differential expression of 25% of the whole transcriptome in cancer cells but only about 0.25% of the transcripts in normal colonic cells.

We also conclude that polyamine synthesis inhibition reversed the changes in the differential expression of transcripts associated with cancer. Specifically, polyamine synthesis inhibition decreased the expression of the TRPV6 and TRPP1 channels, as well as the Orai1 positive modulator SPCA2, probably contributing to decreased Ca^2+^ influx in DFMO-treated tumor cells. In addition, polyamine depletion enhanced the transcription of the plasma membrane PMCA4 Ca^2+^ pump and the mitochondrial channels MCU and VDAC3, thus probably contributing to enhanced Ca^2+^ extrusion in DFMO-treated cells. Collectively, these results may provide a transcriptional basis for the hypothetical calcium remodeling in CRC and its reversal by DFMO. Further research in animal models and/or tumor samples from patients is required to validate our findings.

## Figures and Tables

**Table 1:** Primers used for PCR experiments.

Name	Primers (5′ to 3′)	Predicted Size (pb)
VDAC3	F: TCTATGGCTGGGCTGTGTTG R: ATGTGTGTGCAGCTGGAAGT	137
TRPV6	F: CCTTTGCTGCCTGTGTGAAC R: AGGTTGTACATCTGGCAGGC	151
TRPP1	F: GAGCCTAGACGTGTGGATCG R: GAGCACAGGTCGGTGTTACA	183
TRPC5	F: CCTGGTAGTGCTGCTGAACA R: GGGCTGGGGATGATGTTGAA	165
SPCA2	F: TTCCTCTACTCCGTCCTGGG R: CTCTTGGGGCTGCAACAGTA	191
SEPTIN6	F: TTTGTGAAGCTGCGGGAGAT R: AGCCCATCTCCTCCAGCTTA	112
PMCA4	F: TGGTCAAGTCGCAACTACCC R: GGCAGTCACTAACACCACGA	106
ORMDL3	F: CATCGGTCTCCTCCACATCG R: CACGATGGGTGTGATGGTCA	238
MCU	F: ACTTTGGTGCTATGGGGTGG R: AGGTCCATTTCTGCCTGAGC	296

**Table 2:** Adjusted *p* values of comparisons of resting cytosolic [Ca^2+^], Ca^2+^ store content, and SOCE between experimental conditions shown in Figure 3.

	Adjusted p Value (Red Values Are *p* < 0.05)				
	[Ca^2+^]cyt	Ca^2+^ Store Content		SOCE	
Comparison	Median 1st min	AUC	Max	AUC	Max
HT29 Control − NCM460 Control	5.9962 × 10^−1^	0	2.8816 × 10^−4^	0	0
HT29 DFMO − HT29 Control	5.7354 × 10^−1^	0	0	3.3232 × 10^−2^	7.2647 × 10^−1^
HT29 DFMO − NCM460 Control	9.9997 × 10^−1^	9.8773 × 10^−1^	0	0	0
HT29 DFMO − NCM460 DFMO	1.8581 × 10^−1^	2.4919 × 10^−5^	0	0	0
NCM460 DFMO − HT29 Control	5.3660 × 10^−3^	0	2.6411 × 10^−1^	0	0
NCM460 DFMO − NCM460 Control	1.6650 × 10^−1^	6.4158 × 10^−5^	6.6866 × 10^−2^	9.9962 × 10^−1^	7.7062 × 10^−1^

## Data Availability

All data are available upon request.

## Supplementary Materials

The following supporting information can be downloaded at: https://www.mdpi.com/article/10.3390/cancers15051600/s1, Table S1. Fold changes $\log_{2}\left(Fold\;Change\right)$ (log FC) and corresponding FDR of Figure 4. Table S2. Fold changes $\log_{2}\left(Fold\;Change\right)$ (log FC) and corresponding FDR of Figure 5. Table S3. Fold changes $\log_{2}\left(Fold\;Change\right)$ (log FC) and corresponding FDR of Figure 6. Table S4. Fold changes $\log_{2}\left(Fold\;Change\right)$ (log FC) and corresponding FDR of Figure 7. Table S5. Fold changes $\log_{2}\left(Fold\;Change\right)$ (log FC) and corresponding FDR of Figure 8. Table S6. Fold changes $\log_{2}\left(Fold\;Change\right)$ (log FC) and corresponding FDR of Figure 9. Figure S1. Effects of DFMO treatment on resting Ca^2+^ levels, Ca^2+^ store content and store-operated Ca^2+^ entry (SOCE) in normal NCM460 cells and colon cancer SW480 cells. Cultures of both normal NCM460 and colon cancer SW480 cells were treated with vehicle (a,c) or DFMO (b,d) for 96 h and then loaded with fura2/AM for Ca^2+^ imaging experiments. Resting Ca^2+^ levels were measured be-fore stimulating cells with cyclopiazonic acid (CPA) in Ca^2+^ free media to estimate Ca^2+^ store content. Finally, Ca^2+^ containing media was perfused in the presence of CPA to record SOCE. Representative single cell recordings are shown for cells located in the same optical field. Data are representative of 3 independent experiments for each condition. Figure S2. Statistical analysis of the effects of DFMO treatment on resting Ca^2+^ levels (a), Ca^2+^ store content (b) and store-operated Ca^2+^ entry (SOCE) (c) in NCM460 (blue bars) and SW480 cells (red bars). NCM460 and SW480 were pre-treated with vehicle (control) or DFMO for 96 h and subjected to calcium imaging for analysis of resting Ca^2+^ levels, Ca^2+^ store content and SOCE as shown in Figure 2. Bars plots represent, in original units of response variables (i.e. absolute value, maximum increment or AUC of F340/380 curves), the expected value (bar) and the SEM (error bars). Experimental conditions are grouped into different clusters which are indicated by letters: experimental conditions with different letters mean they are different between them; equal letters mean they are equal, and those experimental conditions with 2 or more letters (e.g., cluster ab) mean they are similar to those groups with any of these letters (i.e., cluster ab is similar both to cluster a and to cluster b). For every experimental condition 8 replicates have been and the total number of cells studied is 76 for NCM460 Control, 88 for SW480 Control, 88 for NCM460 treated with DFMO and 93 for SW480 treated with DFMO. Figure S3. Differences of MCU expression at protein level between HT29 colon cancer cells (Control) colon cancer cells treated with DFMO (DFMO). Protein level expression was carried out by Western-Blot assay, where MCU Expression variable means optical density from MCU normalized to optical density from β-actin. Data set have been analyzed by adjusting a linear model to data, model assumptions have been evaluated, the response variable transformed with Bos-Cox family transformations because of normality assumption was violated, and outlier detection has been carried out by analysis of the cook distance, the residual studentized and the hat values. After transformation, model assumptions were fulfilled: residual normality with the Shapiro-Wilks test (*p* value = 0.1961) and homocedasticity with the Bartlett test (*p* value = 0.8771). The sample size is equal to 6:3 corresponding to HT29 cells without treatment and the other 3 corresponding to HT29 cells treated with DFMO. *p* < 0.01.
