# Supplementary material for: Transcriptional Basis of Ca2+ Remodeling Reversal Induced by Polyamine Synthesis Inhibition in Colorectal Cancer Cells
Source: Cancers (Basel). 2023 Mar 4;15(5):1600. doi: 10.3390/cancers15051600 (PMC10000432; doi:10.3390/cancers15051600)
Supplement: Supplementary file 1 [file cancers-15-01600-s001.zip › cancers-2231288-supplementary.pdf]

Supplementary Tables and Figures

Table S1. Fold changes $\log_2(Fold\ Change)$ (log FC) and corresponding FDR of Figure 5. Red means FDR is lower than 0.05.

	Comparison							
	HT29 vs. NCM460 (Illumina)		HT29 vs. NCM460 (Microarray)		NCM: Control vs. DFMO		HT29: Control vs. DFMO	
Genes	log FC	FDR	log FC	FDR	log FC	FDR	log FC	FDR
**CAV1.1**	5.178E-01	5.011E-01	8.763E-02	8.086E-01	1.113E-01	9.369E-01	1.797E-01	6.739E-01
**CAV1.2**	-1.068E+01	**2.211E-06**	-1.515E+00	**2.432E-04**	5.414E-02	9.738E-01	8.498E-02	8.319E-01
**CAV1.3**	2.099E+00	**6.551E-06**	9.123E-01	**1.577E-02**	3.235E-01	7.246E-01	1.238E-01	7.851E-01
**CAV1.4**	-1.314E+00	1.493E-01	-3.141E-01	2.215E-01	-1.716E-01	8.257E-01	1.135E-01	7.277E-01
**CAV2.1**	-6.354E-01	4.522E-01	2.722E-01	2.145E-01	-2.019E-01	7.237E-01	-3.219E-01	1.969E-01
**CAV2.2**	2.342E-01	8.106E-01	1.725E-01	6.606E-01	1.209E-01	9.385E-01	1.318E-01	7.876E-01
**CAV2.3**	-3.461E+00	**9.392E-03**	-3.151E-01	2.616E-01	1.057E-01	9.267E-01	1.993E-01	5.583E-01
**CAV3.1**	2.278E+00	**2.884E-02**	2.256E-01	4.776E-01	1.265E-01	9.193E-01	-2.623E-02	9.527E-01
**CAV3.2**	-2.822E+00	**1.943E-04**	-4.480E-04	9.989E-01	-9.456E-02	9.283E-01	-3.824E-01	1.880E-01
**CAV3.3**	1.452E+00	9.671E-02	2.393E-02	9.408E-01	-2.769E-01	6.981E-01	-1.549E-01	6.585E-01

Table S2. Fold changes $\log_2(Fold\ Change)$ (log FC) and corresponding FDR of Figure 6. Red means FDR is lower than 0.05.

Genes	HT29 vs. NCM460 (Illumina)		HT29 vs. NCM460 (Microarray)		NCM: Control vs. DFMO		HT29: Control vs. DFMO	
	log FC	FDR	log FC	FDR	log FC	FDR	log FC	FDR
**ORAI1**	2.579E-01	7.525E-02	-4.647E-01	**3.771E-02**	-3.195E-02	9.805E-01	3.131E-01	2.017E-01
**ORAI2**	1.134E+00	**1.724E-06**	3.293E-01	1.384E-01	1.773E-01	7.750E-01	3.148E-01	2.114E-01
**ORAI3**	3.429E-01	1.032E-01	-1.287E-01	5.017E-01	1.949E-01	6.774E-01	-1.415E-01	5.366E-01
**STIM1**	1.397E+00	**1.225E-06**	4.475E-01	**3.063E-02**	-2.056E-01	6.747E-01	4.505E-01	5.052E-02
**STIM2**	7.257E-01	**1.927E-05**	3.276E-02	9.130E-01	-1.493E-01	8.675E-01	8.410E-01	**1.203E-02**
**MS4A12**	5.160E-01	5.042E-01	-3.408E-02	8.902E-01	2.234E-02	9.864E-01	3.094E-02	9.189E-01
**CRACR2A**	-2.391E+00	**6.302E-07**	-3.167E+00	**1.560E-07**	1.977E-01	7.952E-01	5.699E-01	5.839E-02
**STIMATE**	-7.250E-01	**4.438E-02**	-3.065E-01	2.855E-01	-1.269E-04	9.997E-01	1.179E-02	9.792E-01
**ORMDL3**	-2.980E-01	**5.701E-03**	-8.866E-01	**4.248E-03**	3.554E-01	5.588E-01	6.770E-01	**3.550E-02**
**SARAF**	-5.848E-01	**1.066E-04**	-5.397E-01	**4.432E-02**	-2.186E-02	9.881E-01	-4.469E-01	1.311E-01
**EFHB**	-5.036E-01	6.142E-01	-3.482E-01	3.044E-01	-5.381E-01	4.505E-01	1.012E-01	8.189E-01
**MBP**	8.673E-01	**1.090E-05**	3.574E-01	**2.863E-02**	2.049E-01	5.561E-01	1.683E-01	3.538E-01
**SEPTIN1**	-1.701E+00	**3.425E-03**	2.020E-01	3.331E-01	2.174E-01	6.745E-01	7.810E-02	7.698E-01
**SEPTIN2**	7.982E-01	**3.169E-05**	-3.431E-02	9.106E-01	-1.833E-01	8.235E-01	2.582E-01	4.182E-01
**SEPTIN3**	-2.905E+00	**8.880E-06**	-5.630E-01	**2.016E-02**	1.912E-01	7.608E-01	1.603E-01	5.632E-01
**SEPTIN4**	1.387E+00	**3.505E-02**	2.343E-01	1.111E-01	1.349E-01	7.220E-01	-8.828E-02	6.233E-01
**SEPTIN5**	-5.393E-01	4.844E-01	3.322E-01	1.898E-01	6.646E-02	9.506E-01	-2.583E-01	3.826E-01
**SEPTIN6**	-4.570E+00	**3.004E-07**	-4.097E+00	**5.482E-08**	-6.751E-02	9.611E-01	9.010E-01	**1.379E-02**
**SEPTIN7**	3.042E-01	**9.432E-03**	-6.651E-01	**2.198E-02**	1.574E-01	8.634E-01	7.415E-01	**2.435E-02**
**SEPTIN8**	8.330E-01	**8.305E-06**	-1.930E+00	**3.726E-03**	-4.657E-02	9.883E-01	1.339E+00	**4.797E-02**
**SEPTIN9**	1.163E+00	**3.653E-06**	5.643E-01	**1.544E-02**	2.562E-02	9.846E-01	4.537E-01	6.963E-02
**SEPTIN10**	1.280E+00	**1.246E-06**	6.288E-01	**1.725E-02**	-7.009E-01	1.406E-01	-4.211E-01	1.350E-01
**SEPTIN11**	7.746E-01	**3.117E-06**	-7.251E-01	**3.133E-02**	2.931E-01	7.278E-01	9.264E-01	**1.856E-02**

Table S3. Fold changes $\log_2(Fold\ Change)$ (log FC) and corresponding FDR of Figure 7. Red means FDR is lower than 0.05.

Genes	Comparison							
	HT29 vs. NCM460 (Illumina)		HT29 vs. NCM460 (Microarray)		NCM: Control vs. DFMO		HT29: Control vs. DFMO	
	log FC	FDR	log FC	FDR	log FC	FDR	log FC	FDR
**TRPC1**	8.307E-01	2.668E-01	2.307E-01	5.195E-01	-2.793E-02	9.883E-01	-8.272E-01	**4.473E-02**
**TRPC3**			-1.403E-01	5.421E-01	-6.313E-02	9.473E-01	1.360E-01	6.254E-01
**TRPC4**	1.045E+00	1.858E-01	4.635E-01	9.992E-02	3.718E-01	5.498E-01	1.220E-02	9.777E-01
**TRPC5**			5.803E-01	**3.085E-02**	2.791E-01	6.567E-01	-6.531E-01	**3.232E-02**
**TRPC6**	7.612E-01	4.192E-01	-1.772E-01	5.527E-01	-9.890E-02	9.348E-01	6.959E-02	8.576E-01
**TRPC7**	-5.308E+00	**1.203E-05**	-3.487E-01	5.589E-02	-7.148E-02	9.195E-01	-2.432E-01	2.304E-01
**TRPM1**			-1.845E-01	5.493E-01	-1.400E-01	9.036E-01	-2.573E-01	4.744E-01
**TRPM2**	-3.311E+00	**8.102E-06**	-3.986E-01	5.087E-02	1.503E-01	7.957E-01	2.175E-01	3.454E-01
**TRPM3**	-2.874E+00	**2.157E-03**	2.209E-01	4.830E-01	-1.800E-01	8.621E-01	-3.324E-01	3.596E-01
**TRPM4**	8.504E-02	3.651E-01	-2.680E-02	9.024E-01	-1.517E-01	7.845E-01	6.665E-03	9.828E-01
**TRPM5**	-1.530E+00	**2.091E-02**	4.895E-03	9.870E-01	-2.624E-01	7.080E-01	-2.127E-01	5.190E-01
**TRPM6**	-2.370E+00	**1.003E-02**	9.347E-02	7.619E-01	-9.936E-02	9.348E-01	-2.874E-01	4.039E-01
**TRPM7**	5.398E-01	**4.062E-05**	4.446E-01	1.734E-01	-2.501E-01	7.902E-01	-2.206E-01	5.784E-01
**TRPM8**	-3.060E+00	**5.902E-04**	-5.030E-03	9.859E-01	-1.416E-01	8.702E-01	1.967E-01	5.221E-01
**TRPML1**	-1.812E+00	**1.104E-03**	-1.423E+00	**6.834E-05**	7.630E-02	9.385E-01	2.308E-01	4.138E-01
**TRPML2**	-7.489E+00	**1.415E-05**	-2.678E+00	**7.122E-07**	1.360E-01	8.813E-01	1.342E-01	6.841E-01
**TRPML3**	-8.563E+00	**1.557E-05**	-4.811E+00	**9.100E-08**	-3.747E-01	6.831E-01	2.240E-01	6.215E-01
**TRPV1**	-9.464E-01	3.757E-01	-4.933E-01	6.465E-02	-2.306E-01	7.391E-01	3.218E-01	2.831E-01
**TRPV2**	-7.854E-01	4.921E-01	2.116E-01	3.881E-01	-6.120E-02	9.549E-01	-2.376E-01	4.059E-01
**TRPV3**	-5.155E-01	1.354E-01	4.561E-01	1.426E-01	3.000E-01	7.052E-01	-4.578E-02	9.137E-01
**TRPV4**	7.637E-01	4.154E-01	1.256E-01	6.685E-01	-5.589E-02	9.700E-01	-1.529E-01	6.650E-01
**TRPV5**	-4.939E+00	**1.142E-04**	4.657E-01	7.179E-02	-5.483E-02	9.653E-01	-2.982E-01	3.089E-01
**TRPV6**	1.192E+00	**5.980E-03**	2.267E+00	**3.647E-04**	-2.923E-01	8.505E-01	-2.567E+00	**8.441E-04**
**TRPA1**	-1.560E+00	5.378E-02	5.121E-02	8.652E-01	-1.550E-01	8.697E-01	-1.269E-01	7.180E-01
**TRPP1**	-4.127E-01	**2.329E-02**	6.374E-01	**2.208E-02**	-2.567E-01	7.025E-01	-1.032E+00	**4.269E-03**
**TRPP2**	2.311E-01	**2.191E-02**	-8.599E-01	5.782E-02	-5.984E-02	9.823E-01	8.684E-01	8.653E-02
**TRPP3**	-2.497E+00	**9.610E-03**	-6.127E-02	8.525E-01	-1.714E-01	8.675E-01	-2.625E-02	9.512E-01
**TRPP5**	-2.488E+00	**2.143E-02**	-5.338E-01	1.098E-01	-2.881E-01	7.431E-01	-8.713E-02	8.414E-01

Table S4. Fold changes $\log_2(Fold\ Change)$ (log FC) and corresponding FDR of Figure 8. Red means FDR is lower than 0.05.

Genes	Comparison							
	HT29 vs. NCM460 (Illumina)		HT29 vs. NCM460 (Microarray)		NCM: Control vs. DFMO		HT29: Control vs. DFMO	
	log FC	FDR	log FC	FDR	log FC	FDR	log FC	FDR
**IP3R1**	1.628E+00	**2.104E-04**	3.113E-01	2.085E-01	7.896E-01	1.011E-01	4.341E-01	1.242E-01
**IP3R2**	-1.525E+00	**2.019E-06**	-1.261E+00	**4.091E-03**	-7.767E-02	9.686E-01	-1.138E-01	8.211E-01
**IP3R3**	1.091E+00	**3.814E-05**	9.185E-01	**5.396E-03**	-3.685E-01	5.770E-01	-3.125E-01	3.614E-01
**RYR1**	1.292E+00	9.894E-02	-2.766E-02	9.274E-01	-1.960E-01	8.012E-01	3.680E-02	9.196E-01
**RYR2**	-7.876E+00	**1.818E-05**	-1.716E+00	**1.591E-03**	6.301E-01	5.244E-01	-5.017E-02	9.364E-01
**RYR3**	1.717E+00	**5.988E-04**	-1.813E-01	4.607E-01	2.717E-01	6.418E-01	3.851E-02	9.078E-01

Table S5. Fold changes $\log_2(Fold\ Change)$ (log FC) and corresponding FDR of Figure 9. Red means FDR is lower than 0.05.

	Comparison							
	HT29 vs. NCM460 (Illumina)		HT29 vs. NCM460 (Microarray)		NCM: Control vs. DFMO		HT29: Control vs. DFMO	
**Genes**	**log FC**	**FDR**	**log FC**	**FDR**	**log FC**	**FDR**	**log FC**	**FDR**
**PMCA1**	1.199E+00	**2.292E-06**	1.317E+00	**3.284E-04**	1.349E-01	8.952E-01	1.583E-01	6.439E-01
**PMCA2**			3.381E-01	2.363E-01	6.918E-02	9.573E-01	-2.175E-01	5.284E-01
**PMCA3**	-1.842E+00	**1.352E-02**	2.445E-01	3.162E-01	2.218E-01	7.273E-01	-7.059E-02	8.248E-01
**PMCA4**	-2.342E+00	**3.936E-07**	-1.788E+00	**1.370E-05**	-7.213E-02	9.464E-01	9.198E-01	**5.665E-03**
**SERCA1**	-2.349E-01	6.386E-01	1.340E-01	7.093E-01	1.869E-01	8.738E-01	-1.083E-01	8.089E-01
**SERCA2**	2.410E-01	**3.740E-02**	2.198E-01	2.672E-01	6.825E-02	9.333E-01	8.515E-02	7.354E-01
**SERCA3**	2.799E-01	**1.280E-02**	1.548E-01	5.504E-01	-1.218E-01	8.988E-01	1.352E-01	6.706E-01
**NCX1**	1.870E-01	4.511E-01	2.310E-01	3.670E-01	2.186E-01	7.502E-01	2.982E-01	3.119E-01
**NCX2**	3.251E+00	**8.235E-04**	7.204E-01	**3.893E-03**	-3.093E-01	5.141E-01	-6.608E-02	8.161E-01
**NCX3**			4.335E-01	5.988E-02	1.718E-01	7.924E-01	-1.389E-01	6.156E-01
**SPCA1**	-6.110E-01	**4.334E-04**	-5.977E-01	**8.779E-03**	7.924E-02	9.248E-01	2.403E-01	3.169E-01
**SPCA2**	9.951E-02	6.008E-01	5.729E-01	**5.949E-03**	-5.428E-01	1.328E-01	-9.795E-01	**9.028E-04**

Table S6. Fold changes $\log_2(Fold\ Change)$ (log FC) and corresponding FDR of Figure 10. Red means FDR is lower than 0.05.

Genes	Comparison							
	HT29 vs. NCM460 (Illumina)		HT29 vs. NCM460 (Microarray)		NCM: Control vs. DFMO		HT29: Control vs. DFMO	
	log FC	FDR	log FC	FDR	log FC	FDR	log FC	FDR
**MCU**	9.436E-01	**1.461E-05**	9.348E-01	**9.154E-04**	-6.143E-03	9.944E-01	5.891E-01	**2.942E-02**
**MICU1**	6.902E-01	**4.420E-05**	2.180E-01	4.172E-01	-9.223E-02	9.332E-01	4.813E-01	1.138E-01
**MICU2**	-7.924E-01	**2.762E-05**	-1.547E+00	**5.842E-04**	-2.880E-01	7.676E-01	5.772E-01	1.496E-01
**MICU3**			-3.598E-01	1.174E-01	1.764E-01	7.863E-01	2.020E-01	4.551E-01
**MCUR1**	9.974E-01	**3.089E-05**	5.618E-02	8.396E-01	4.967E-02	9.712E-01	5.574E-02	8.729E-01
**EMRE**	1.826E-01	**4.656E-02**	-9.551E-02	6.629E-01	3.506E-01	4.315E-01	3.929E-01	1.046E-01
**MCUb**	-2.140E+00	**6.875E-06**	-5.020E+00	**4.695E-05**	-3.784E-01	9.036E-01	1.345E+00	1.452E-01
**VDAC1**	-1.687E+00	**2.233E-07**	-1.357E+00	**7.975E-05**	3.181E-02	9.823E-01	4.201E-01	1.165E-01
**VDAC2**	2.716E-01	**1.346E-03**	2.793E-01	1.667E-01	-4.752E-02	9.597E-01	-1.400E-01	5.668E-01
**VDAC3**	-1.411E+00	**2.284E-05**	-2.574E+00	**7.112E-05**	8.104E-02	9.717E-01	1.022E+00	**4.564E-02**
**NCLX**	-1.900E+00	**9.815E-05**	-9.316E-01	**2.609E-04**	-1.123E-01	8.535E-01	1.240E-01	5.921E-01

FIGURE S1

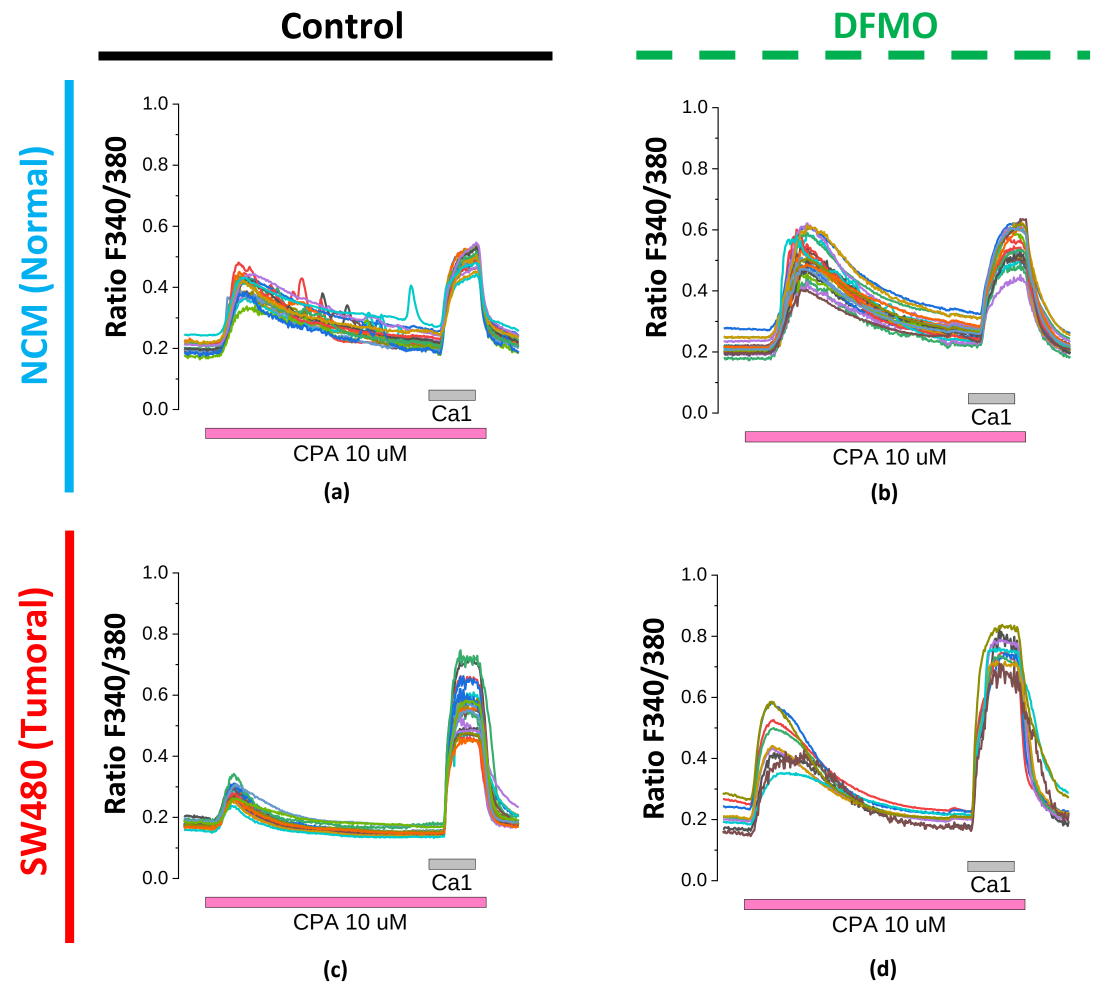

Figure S1. Effects of DFMO treatment on resting Ca^{2+} levels. Ca^{2+} store content and store-operated Ca^{2+} entry (SOCE) in normal NCM460 cells and colon cancer SW480 cells. Cultures of both normal NCM460 and colon cancer SW480 cells were treated with vehicle (a.c) or DFMO (b.d) for 96 h and then loaded with fura2/AM for Ca^{2+} imaging experiments. Resting Ca^{2+} levels were measured before stimulating cells with cyclopiazonic acid (CPA) in Ca^{2+} free media to estimate the Ca^{2+} store content. Finally. Ca^{2+} containing media was perfused in the presence of CPA to recording SOCE. Representative single cell recordings are shown for cells located in the same optical field. Data are representative of 3 independent experiments for each condition.

FIGURE S2

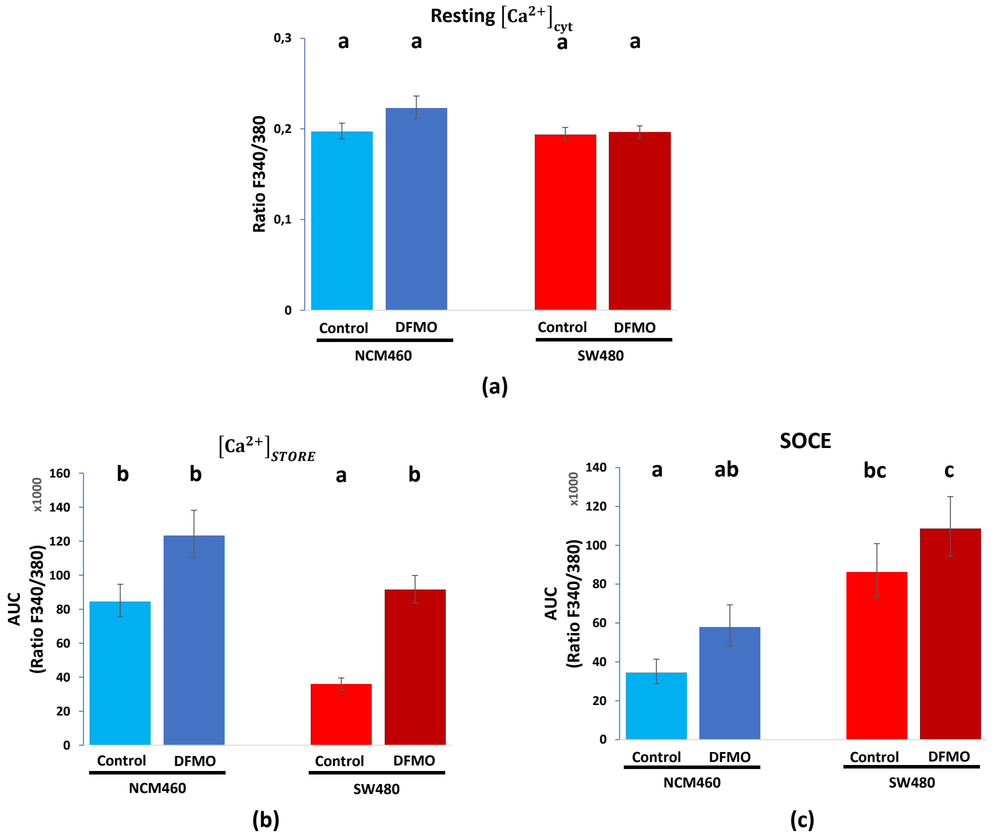

Figure S2. Statistical analysis of the effects of DFMO treatment on resting Ca^{2+} levels (a). Ca^{2+} store content (b) and store-operated Ca^{2+} entry (SOCE) (c) in NCM460 (blue bars) and SW480 cells (red bars). NCM460 and SW480 were pre-treated with vehicle (control) or DFMO for 96 h and subjected to calcium imaging for analysis of resting Ca^{2+} levels. Ca^{2+} store content and SOCE as shown in Fig. 2. Bars plots represent. in original units of response variables (i.e. absolute value. maximum increment or AUC of F340/380 curves). the expected value (bar) and the SEM (error bars). Experimental conditions are grouped into different clusters which are indicated by letters: experimental conditions with different letters mean they are different between them; equal letters mean they are equal and those experimental conditions with 2 or more letters (e.g. cluster ab) mean they are similar to those groups with any of these letters (i.e. cluster ab is similar both to cluster a and to cluster b). For every experimental condition 8 replicates have been used and the total number of cells studied is 76 for NCM460 Control. 88 for SW480 Control. 88 for NCM460 treated with DFMO and 93 for SW480 treated with DFMO.

FIGURE S3.

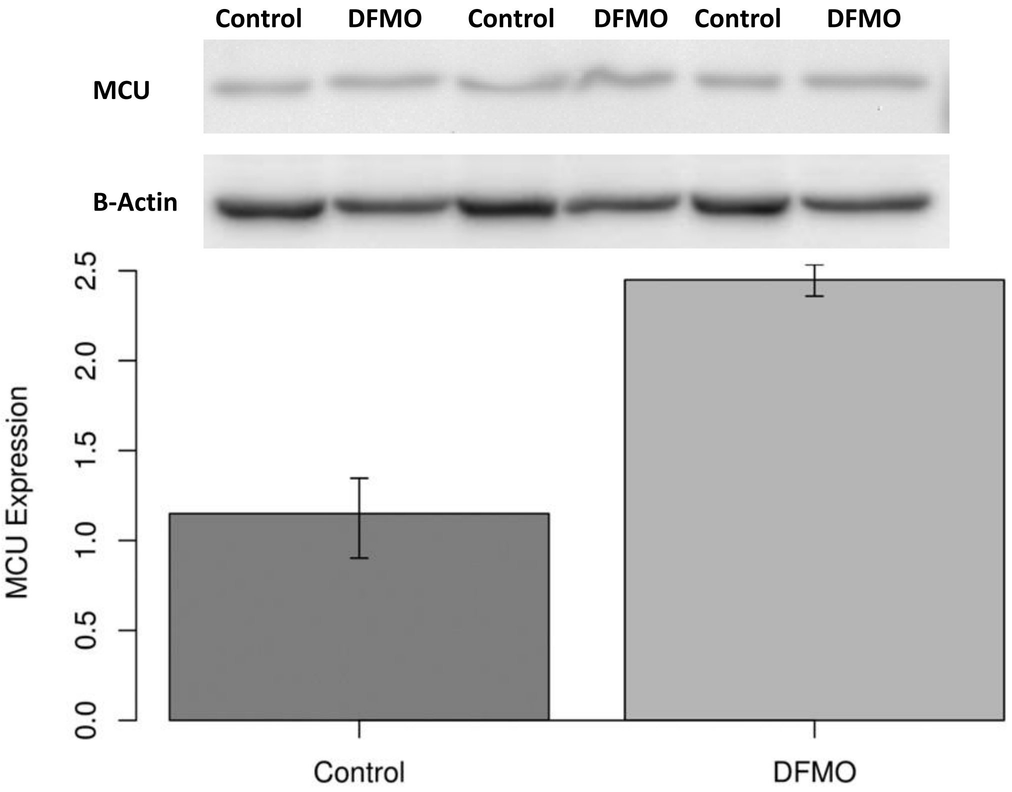

Figure S3. Differences of MCU expression at protein level between HT29 colon cancer cells (Control) and colon cancer cells treated with DFMO (DFMO). Protein level expression was carried out by Western-Blot assay where MCU Expression variable means optical density from MCU normalized to optical density from β-actin. Data set have been analyzed by adjusting a linear model to data. Model assumptions have been evaluated. The response variable transformed with Bos-Cox family transformations because of normality assumption was violated and outlier detection has been carried out by analysis of the cook distance. The residual studentized and the hat values. After transformation, model assumptions were fulfilled: residual normality with the Shapiro-Wilks test (p value = 0.1961) and homocedasticity with the Bartlett test (p value = 0.8771). The sample size is equal to 6: 3 corresponding to HT29 cells without treatment and the other 3 corresponding to HT29 cells treated with DFMO. $p < 0.01$.
